# Expression Profile of MicroRNAs in Young Stroke Patients

**DOI:** 10.1371/journal.pone.0007689

**Published:** 2009-11-02

**Authors:** Kay Sin Tan, Arunmozhiarasi Armugam, Sugunavathi Sepramaniam, Kai Ying Lim, Karolina Dwi Setyowati, Chee Woon Wang, Kandiah Jeyaseelan

**Affiliations:** 1 Departments of Medicine and Molecular Medicine, University of Malaya, Kuala Lumpur, Malaysia; 2 Department of Biochemistry, Yong Loo Lin School of Medicine, National University of Singapore, Singapore, Singapore; University of Nebraska, United States of America

## Abstract

**Background:**

The methods currently available for diagnosis and prognosis of cerebral ischaemia still require further improvements. Micro-RNAs (small non-coding RNAs) have been recently reported as useful biomarkers in diseases such as cancer and diabetes. We therefore carried out microRNA (miRNA) profiling from peripheral blood to detect and identify characteristic patterns in ischaemic stroke.

**Methods/Principal Findings:**

The ischaemic stroke patients aged between 18–49 years, characterized based on World Health Organization clinical criteria were further classified according to TOAST classification, **a)** Large-vessel atherosclerosis [n = 8] **b)** Small-vessel disease [n = 3] **c)** Cardioembolism [n = 5] **d)** Undetermined cause [n = 3]. The patients' functional status at the time of blood sampling (at the outpatient clinics) was evaluated with the modified Rankin Scale (mRS). Blood samples from normal (n = 5) individuals were used as controls. Total RNA extracted from whole blood was subjected to miroRNA profiling and real-time PCR analysis.

miRNAs that are implicated in the endothelial/vascular function, erythropoiesis, angiogenesis and neural function showed differential expression profile as compared to the normal control. Interestingly, miRNAs that are involved in hypoxic conditions have also been found in our miRNA profiles.

**Conclusion:**

We demonstrate that the peripheral blood miRNAs and their profiles can be developed as biomarkers in diagnosis and prognosis of cerebral ischaemic stroke. The dysregulated miRNAs have been detectable even after several months from the onset of stroke in what is usually regarded as neurologically stable patients.

## Introduction

MicroRNAs (miRNAs) are tiny (∼19–23 nt) non-coding RNA molecules that are currently being recognized as endogenous physiological regulators of gene expression. These small RNAs are capable of controlling gene expression either by repression of translation/transcription (RNAi)[1] or by activation (RNAa) of transcription[2]. MiRNAs are also known to play important roles in many physiological and pathological processes, including tumorigenesis[3], proliferation[4], hematopoiesis[5], metabolism[6], immune function[7], epigenetics and neurodegenerative diseases[8]. MiRNAs have also been found to be useful in identifying the etiology of lymphoma[9] and progression of certain neurological diseases[10]. However, only few reports are available on the roles of miRNAs in cerebral ischemia/brain injury in animal models[11]–[13]. Using rodent models for ischemic stroke (MCAo), we have shown that miRNAs are temporally regulated during progression/reperfusion of cerebral ischemia and miRNAs in total blood could be used as diagnostic markers. Similarly, in traumatic brain injury (murine model) temporal regulation of miRNA expression observed has been correlated to several biological processes underlying the brain injury[12]. Moreover Chen et al[14] have demonstrated that serum/plasma miRNAs derived from various tissues/organs are stable and resistant to nuclease digestion. Expression levels of miRNAs in blood have been found to be reproducible and indicative of the disease state[14]. Thus we propose that specific signatures of blood miRNA could be obtained from total blood samples and can be used in the identification of biomarkers for diagnosis, prognosis or even etiology of a disease. In this study, using the blood samples obtained from young ischemic stroke patients (18 to 49 years) we have shown that besides the disease progression, the stroke subtype could also be identified via the miRNA profiles.

## Results

### MiRNA Profiles

**Figure 1:**
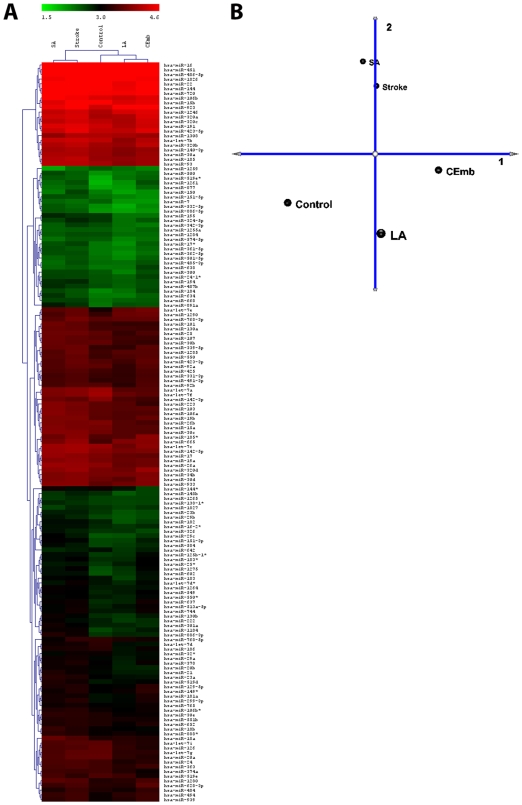
microRNA profile data. **(a)** MicroRNA profiles: For normal control, all stroke samples pooled (stroke, n = 19), small artery (SA, n = 3), large artery (LA, n = 8) and cardioembolic (CEmb, n = 5) stroke. The hierachical clustering was carried out for both the samples as well as the miRNAs. The average signal intensities for each significantly expressed miRNA (one way ANOVA, p value<0.05) was log10 transformed and the values have been used for the construction the tree. **(b)**. Principal Component Analysis (PCA) were carried out on the same set of data. The hierachical clustering and PCA were constructed using the using the TM4 software [29].

The profiles demonstrate that microRNAs can be detected in total peripheral blood in human as was demonstrated for rat's blood[11]. The heat maps generated also indicate that the microRNAs are differentially expressed between normal and stroke subjects (Figure 1a). Differential expression of miRNAs could also be observed among the samples representing different stroke subtypes. Many miRNAs that are poorly expressed in normal subjects have been found to be highly expressed in stroke samples. All the miRNAs that are statisticlly significant and differentially regulated in all stroke samples are listed in Table S1. Stem-loop real-time PCR results on selected miRNAs (miR-16, 126, -144, -21, -223 and -320a; Table 1) also have been found to be consistent with the expression patterns observed by miRNA profiling.

**Table 1:** Validation of microarray data.

**microRNA microarray data (fold change)**											
**miRNA**	**Stroke**	**Stroke mRS<2**	**Stroke mRS>2**	**LA**	**LA mRS<2**	**LA mRS>2**	**CEmb**	**CEmb mRS<2**	**CEmb mRS>2**	**SA mRS<2**	**UND mRS<2**
**hsa-miR-126**	0.79*±0.02*	0.31*±0.01*	0.75*±0.05*	0.50*±0.07*	0.16*±0.02*	0.84*±0.08*	0.37*±0.03*	0.20*±0.01*	0.53*±0.05*	0.71*±0.05*	0.92*±0.14*
**hsa-miR-144**	1.35*±0.08*	0.87*±0.07*	1.61*±0.07*	0.85*±0.02*	0.42*±0.06*	1.28*±0.01*	1.17*±0.05*	0.65*±0.09*	1.70*±0.03*	1.70*±0.03*	1.57*±0.00*
**hsa-miR-16**	1.02*±0.02*	0.62*±0.08*	1.03*±0.03*	0.67*±0.02*	0.30*±0.03*	1.03*±0.03*	0.74*±0.02*	0.46*±0.03*	1.02*±0.04*	1.03*±0.03*	1.01*±0.04*
**hsa-miR-21**	1.34*±0.14*	0.58*±0.15*	1.28*±0.15*	0.75*±0.00*	0.17*±0.17*	1.33*±0.02*	0.75*±0.12*	0.35*±0.12*	1.15*±0.12*	1.72*±0.15*	1.79*±0.20*
**hsa-miR-223**	1.44*±0.04*	0.88*±0.08*	1.42*±0.02*	0.93*±0.06*	0.25*±0.01*	1.61*±0.08*	0.57*±0.03*	0.35*±0.06*	0.80*±0.02*	1.71*±0.04*	1.72*±0.11*
**hsa-miR-320a**	2.07*±0.11*	1.19*±0.10*	2.60*±0.10*	1.10*±0.01*	0.70*±0.11*	1.50*±0.03*	2.18*±0.05*	1.57*±0.09*	2.80*±0.03*	1.75*±0.12*	1.35*±0.11*
**microarray results validated by stem-loop real-time PCR (relative expression)**											
**miRNA**	**Stroke**	**Stroke mRS<2**	**Stroke mRS>2**	**LA**	**LA mRS<2**	**LA mRS>2**	**CEmb**	**CEmb mRS<2**	**CEmb mRS>2**	**SA mRS<2**	**UND mRS<2**
**hsa-miR-126**	0.81*±0.06*	0.45*±0.02*	1.11*±0.02*	0.85*±0.02*	0.35*±0.05*	1.05*±0.03*	0.54*±0.09*	0.32*±0.08*	0.82*±0.05*	0.94*±0.08*	1.18*±0.05*
**hsa-miR-144**	2.46*±0.08*	1.53*±0.04*	2.73*±0.10*	1.55*±0.01*	0.78*±0.09*	2.53*±0.06*	2.09*±0.08*	1.24*±0.05*	3.14*±0.03*	3.43*±0.04*	1.96*±0.02*
**hsa-miR-16**	1.86*±0.06*	1.09*±0.03*	1.69*±0.05*	1.17*±0.02*	0.53*±0.01*	1.64*±0.05*	1.27*±0.01*	0.73*±0.07*	1.75*±0.02*	1.86*±0.07*	1.59*±0.03*
**hsa-miR-21**	2.40*±0.07*	1.07*±0.01*	2.27*±0.08*	1.35*±0.01*	0.32*±0.01*	2.29*±0.00*	1.29*±0.03*	0.68*±0.08*	1.95*±0.01*	3.16*±0.04*	3.45*±0.07*
**hsa-miR-223**	2.01*±0.02*	1.01*±0.02*	2.47*±0.06*	1.23*±0.03*	0.47*±0.02*	2.42*±0.05*	0.78*±0.00*	0.58*±0.03*	1.3*±0.01*	2.52*±0.03*	2.76*±0.05*
**hsa-miR-320a**	3.60*±0.07*	2.04*±0.00*	4.21*±0.06*	1.83*±0.01*	1.30*±0.06*	2.58*±0.04*	3.09*±0.04*	2.56*±0.06*	4.84*±0.04*	3.16*±0.06*	2.40*±0.05*

Six microRNAs were randomly selected from the array results and their expression level was quantitated using the stem-loop PCR. The relative expression values are shown with ±SEM. Stroke (all stroke cases, n = 19; StrokemRS<2 (n = 15), StrokemRS>2 (n = 4); Small artery stroke (SA) mRS<2 (n = 3), Large artery stroke (LA; n = 8) [LAmRS<2 (n = 6), LAmRS>2 (n = 2)] and Cardioembolic stroke (CEmb; n = 5) [mRS<2 (n = 3), mRS>2 (n = 2)], stroke due to undetermined cause (UND, n = 3). Sample identification is as listed in Table S1.

Hierachical clustering (Figure 1a) showed two main branches. All stroke samples that have been pooled together, (n = 19) and designated as “stroke” and the small artery stroke sample (SA; n = 3) formed one cluster. The large artery stroke samples (LA; n = 8) and cardioembolic stroke samples (CEmb; n = 5) formed another cluster. The control samples (control; n = 5) were found to form a separate cluster from that of LA and CEmb. Principal component analysis (PCA; Figure 1b) showed that among the five sample groups (control, stroke, LA, CEmb and SA), LA was clustered with CEmb and SA was clustered with Stroke as was seen with hierachical clustering. Despite these similarities, the samples remained significantly different from each other.

### Expression of MiRNAs that Are Significantly Affected by Stroke

Among the 836 microRNAs (mirBase version 11.0; Sanger Database), present on the array chip, 157 microRNAs have been found to be differentially regulated (one way ANOVA, p<0.05) across stroke samples and the subtypes (LA, SA, CEmb, UND; Table S1). Among the 157 microRNAs identified for stroke samples (n = 19), 138 miRNAs have been found to be highly expressed (upregulated; fold change >1.0) and 19 miRNAs have been found to be poorly expressed (downregulated; fold change <1.0). Within these 19 poorly expressed microRNAs, we could observe 8 miRNAs (hsa-let-7f, miR-126, -1259, -142-3p, -15b, -186, -519e, -768-5p) to be poorly expressed across the three subtypes of stroke (LA, SA and CEmb) that were examined. Similarly, among the highly expressed (138) miRNAs that were observed for stroke, 17 microRNAs (hsa-let-7e, miR-1184, -1246, -1261, -1275, -1285, -1290, -181a, -25*, -513a-5p, -550, -602, -665, -891a, -933, -939, -923) can also be identified as highly expressed in the subtypes (Table 2).

**Table 2:** microRNAs that show similar expression among the various stroke samples.

miRNA	Stroke	Stroke mRS<2	Stroke mRS>2	LA	LA mRS<2	LA mRS>2	CEmb	Cemb mRS<2	Cemb mRS>2	SA	UND
**hsa-let-7e**	3.02±0.11	2.290.00	3.62±0.09	3.20±0.02	3.12±0.04	3.03±0.09	3.83±0.00	3.79±0.05	3.87±0.04	2.60±0.05	1.56±0.00
**hsa-let-7f**	0.69±0.05	0.30±0.06	0.70±0.07	0.45±0.08	0.15±0.07	0.75±0.08	0.37±0.09	0.20±0.09	0.54±0.10	0.60±0.08	0.97±0.07
**hsa-miR-1184**	3.09±0.04	1.78±0.08	2.18±0.02	1.43±0.03	1.19±0.08	1.53±0.02	1.92±0.06	1.33±0.05	2.51±0.11	3.11±0.01	2.21±0.06
**hsa-miR-1246**	1.66±0.00	1.44±0.01	1.68±0.00	1.78±0.05	2.25±0.09	1.29±0.03	2.09±0.05	2.07±0.00	2.10±0.09	1.29±0.05	1.67±0.08
**hsa-miR-1259**	0.71±0.06	0.17±0.18	0.92±0.05	0.74±0.08	0.62±0.07	0.85±0.19	0.54±0.20	0.22±0.92	0.78±0.02	0.34±0.03	0.63±0.01
**hsa-miR-126**	0.79±0.02	0.31±0.01	0.75±0.05	0.50±0.07	0.16±0.02	0.84±0.08	0.37±0.03	0.20±0.01	0.53±0.05	0.71±0.05	0.92±0.14
**hsa-miR-1261**	3.95±0.07	1.81±0.04	5.52±0.05	1.82±0.06	1.51±0.06	1.83±0.06	4.02±0.02	1.35±0.21	6.65±0.07	2.87±0.07	3.08±0.08
**hsa-miR-1275**	3.06±0.04	2.27±0.02	3.01±0.02	1.51±0.05	1.63±0.03	1.24±0.07	2.71±0.06	2.51±0.04	2.91±0.08	2.32±0.04	3.36±0.03
**hsa-miR-1285**	1.87±0.06	1.22±0.06	1.68±0.07	1.34±0.01	1.54±0.00	1.14±0.03	1.37±0.02	1.04±0.02	1.70±0.03	1.30±0.04	1.39±0.01
**hsa-miR-1290**	2.01±0.03	1.70±0.04	1.98±0.02	2.14±0.01	2.71±0.01	1.57±0.05	2.27±0.05	2.20±0.05	2.33±0.05	1.37±0.01	2.27±0.01
**hsa-miR-142-3p**	0.59±0.02	0.28±0.01	0.69±0.01	0.43±0.08	0.18±0.03	0.68±0.10	0.37±0.03	0.15±0.03	0.60±0.05	0.70±0.01	0.86±0.06
**hsa-miR-15b**	0.94±0.01	0.53±0.05	0.93±0.07	0.59±0.04	0.48±0.09	0.70±0.00	0.70±0.08	0.59±0.06	0.81±0.09	0.65±0.06	0.66±0.11
**hsa-miR-181a**	1.64±0.04	1.56±0.06	1.74±0.06	1.14±0.04	1.06±0.02	1.22±0.05	1.89±0.03	1.64±0.02	2.15±0.04	1.76±0.03	2.26±0.06
**hsa-miR-186**	0.84±0.03	0.48±0.03	0.81±0.01	0.44±0.03	0.21±0.06	0.68±0.02	0.67±0.07	0.52±0.00	0.81±0.11	0.83±0.04	0.74±0.12
hsa-miR-25*	1.64±0.07	1.21±0.04	1.91±0.03	1.48±0.09	1.27±0.07	1.66±0.21	2.18±0.20	1.26±0.04	3.07±0.26	1.62±0.05	1.45±0.17
**hsa-miR-513a-5p**	2.04±0.00	1.30±0.03	2.01±0.02	1.29±0.05	1.30±0.00	1.28±0.09	1.89±0.01	1.62±0.00	2.16±0.01	1.58±0.00	1.76±0.01
**hsa-miR-519e**	0.63±0.05	0.59±0.00	0.90±0.02	0.78±0.16	0.58±0.06	0.97±0.21	0.43±0.03	0.30±0.07	0.57±0.00	0.93±0.06	0.76±0.08
**hsa-miR-550**	1.81±0.09	1.06±0.10	1.91±0.09	1.32±0.04	1.29±0.12	1.33±0.04	1.52±0.12	1.09±0.12	1.95±0.13	1.49±0.10	1.36±0.15
**hsa-miR-602**	2.48±0.03	1.83±0.08	3.06±0.03	1.47±0.01	1.37±0.08	1.56±0.10	2.57±0.06	1.94±0.02	3.19±0.08	2.45±0.10	2.46±0.04
**hsa-miR-665**	3.02±0.10	1.81±0.10	3.26±0.10	2.33±0.03	2.26±0.07	2.39±0.11	2.67±0.02	2.39±0.04	2.96±0.01	2.05±0.06	1.57±0.05
**hsa-miR-768-5p**	0.97±0.06	0.42±0.09	1.05±0.10	0.75±0.01	0.67±0.09	0.84±0.05	0.67±0.14	0.42±0.06	0.93±0.18	0.52±0.08	0.80±0.17
**hsa-miR-891a**	2.48±0.12	1.85±0.05	2.10±0.17	1.61±0.17	2.00±0.15	1.42±0.19	1.61±0.18	1.12±0.13	2.09±0.21	3.55±0.14	1.74±0.19
**hsa-miR-923**	2.41±0.02	2.12±0.06	2.34±0.02	2.08±0.10	2.42±0.06	1.56±0.17	2.68±0.02	2.85±0.03	2.50±0.07	1.58±0.01	2.83±0.05
**hsa-miR-933**	1.83±0.02	1.32±0.01	1.75±0.00	1.31±0.00	1.17±0.00	1.46±0.00	1.63±0.00	1.33±0.03	1.92±0.02	1.84±0.03	1.44±0.02
**hsa-miR-939**	2.15±0.02	1.29±0.02	2.47±0.06	-	1.12±0.02	1.71±0.07	1.96±0.03	1.45±0.00	2.47±0.05	1.59±0.01	1.63±0.04

Value expressed as fold change ±SEM of each sample is based on pooled sample of individual stroke subtype. The signal log ratio (stroke/normal control) of the averaged signal values were log2 transformed to obtain the fold change. mRS: modified Rankin Score; LA: large artery stroke; SA: small artery stroke; CEmb: cardioembolic stroke; UND: undetermined cause.

### MiRNA Patterns in Small Artery (SA) *vs* Large Artery (LA) Stroke

The miRNA profile of small artery (SA) stroke samples showed a distinctly different pattern from that of the large artery (LA) strokes samples (Figure 1a). Seventy nine (79) miRNAs could be identified as differentially regulated among these two subtypes (Table 3). Of these, in the small artery (SA) stroke samples, 77 constituted the highly expressed (fold change >1) microRNAs (Table 3). Seven of them (hsa-let-7d*, miR-16, -26b, -150, -374a, -320c, -652) were found to be expressed at the basal level (fold change 1.0–1.04) and one (miR-768-3p) was found to be poorly expressed (fold change 0.92±0.06). An opposite trend in expression was observed in the large artery (LA) stroke samples. Of the total 79 miRNAs, 77 were downregulated and one (miR-939) was undetectable and the other (miR-768-3p) was upregulated. It is interesting to note that the microRNAs that have been found to be upregulated in LA have also been found to be highly expressed in all stroke cases (n = 19). Of the highly upregulated (fold change>1.5) miRNAs, 7 miRNAs (hsa-miR-130b, -29b, -301a, -339-5p, -532-5p, -634, 886-5p) showed more than 2 fold change in SA samples.

**Table 3:** miRNA expression pattern in large artery (LA) and small artery (SA) stroke.

hsa-miRNA	Stroke	LA	SA	hsa-miRNA	Stroke	LA	SA	hsa-miRNA	Stroke	LA	SA
let-7b	1.23	0.51	1.13	miR-18b	1.46	0.87	1.90	miR-320b	1.52	0.86	1.26
let-7c	1.18	0.55	1.10	miR-191	1.47	0.70	1.34	miR-320c	1.39	0.78	1.04
let-7d*	1.59	0.77	1.02	miR-194	1.79	0.87	1.64	miR-320d	1.56	0.87	1.28
miR-101	1.54	0.83	1.98	miR-195	1.06	0.45	1.32	miR-331-3p	1.44	0.89	1.15
miR-103	1.31	0.84	1.64	miR-19a	1.41	0.82	1.23	**miR-339-5p**	2.26	0.98	**2.08**
miR-106a	1.49	0.84	1.77	miR-19b	1.65	0.88	1.62	miR-361-5p	1.58	0.88	1.80
miR-106b	1.42	0.77	1.77	miR-20b	1.29	0.69	1.37	miR-362-5p	1.78	0.75	1.73
miR-106b*	1.42	0.80	1.37	miR-21	1.34	0.75	1.72	miR-363	1.24	0.67	1.10
miR-107	1.17	0.76	1.37	miR-222	1.31	0.67	1.70	miR-374a	0.93	0.66	1.00
miR-1265	1.36	0.99	1.13	miR-223	1.44	0.93	1.71	miR-378	1.43	0.63	1.22
miR-130a	1.56	0.99	1.70	miR-23a	1.63	0.86	1.78	miR-425	1.29	0.82	1.19
**miR-130b**	1.78	0.89	**2.33**	miR-23b	1.37	0.67	1.39	miR-487b	1.27	0.76	1.62
miR-138-1*	1.20	0.93	1.43	miR-24	1.23	0.58	1.17	miR-501-5p	1.52	0.74	1.20
miR-140-3p	1.29	0.72	1.12	miR-24-1*	1.19	0.81	1.24	**miR-532-5p**	1.65	0.66	**2.10**
miR-142-5p	1.42	0.80	1.24	miR-25	1.24	0.56	1.23	miR-550*	1.37	0.85	1.27
miR-144	1.35	0.85	1.70	miR-26a	1.17	0.58	1.28	miR-551b	1.12	0.89	1.05
miR-144*	0.69	0.60	1.07	miR-26b	0.97	0.57	1.00	miR-628-3p	1.67	0.96	1.33
miR-150	2.22	0.61	1.01	miR-29a	1.40	0.75	1.34	**miR-634**	1.70	0.94	**2.72**
miR-151-5p	1.47	0.75	1.56	**miR-29b**	1.58	0.84	**2.03**	miR-652	1.14	0.71	1.01
miR-15a	1.07	0.63	1.21	miR-300	1.28	0.56	1.31	miR-720	1.63	0.96	1.27
miR-16	1.02	0.67	1.03	**miR-301a**	1.56	0.97	**2.05**	miR-744	1.84	0.98	1.29
miR-16-2*	1.42	0.84	1.38	miR-30a	1.30	0.72	1.33	miR-765	1.33	0.91	1.17
miR-17	1.26	0.72	1.48	miR-30b	0.98	0.59	1.07	miR-768-3p	1.49	1.13	0.92
miR-17*	2.02	0.73	1.63	miR-30c	1.29	0.66	1.30	**miR-886-5p**	2.10	0.82	**2.36**
miR-182	1.35	0.48	1.37	miR-30d	1.48	0.77	1.29	miR-93	1.29	0.70	1.33
miR-183	1.32	0.54	1.22	miR-30e	1.16	0.71	1.21	miR-939	2.15	-	1.59
miR-185	1.78	0.93	1.54								

microRNAs that shows opposite expression between the two subtypes (LA & SA) are listed. miRNAs that are expressed more than 2 fold change are in **bold letters**. ±SEM values are as listed in Table S1.

### Undetermined Stroke *vs* Stroke Subtypes

**Figure 2:**
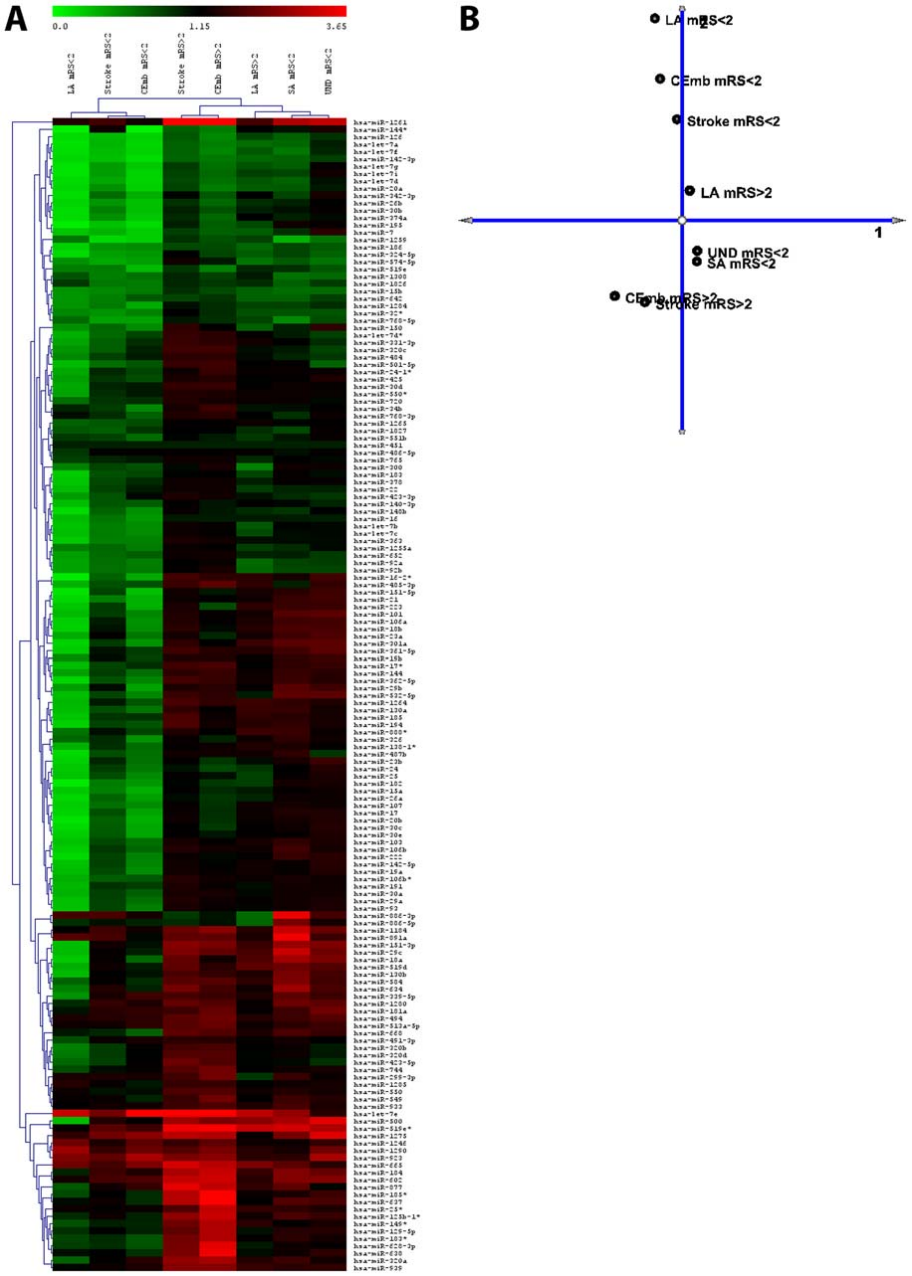
MicroRNA profiles for the stroke subtypes based on their mRS scores. **(a)** The PCA analysis carried out on the pooled stroke samples. **(b)** Hierachical clustering was carried out for both the samples as well as the miRNAs as described. (Stroke mRS<2 (n = 15), Stroke mRS>2 (n = 4); Small artery (SA) mRS<2 (n = 3), Large artery (LA) mRS<2 (n = 6), Large artery (LA) mRS>2 (n = 2) and Cardioembolic (CEmb) mRS<2 (n = 3), Cardioembolic (CEmb) mRS>2 (n = 2) and undetermined cause (UND) mRS<2 (n = 3).

Hierachical clustering analysis on the microRNA profiles observed among the different subtypes (Figure 2a) showed that undetermined stroke samples (n = 3, mRS<2) resembled the profile of small artery stroke (SA, n = 3, mRS<2). PCA (Figure 2b) also showed that the SA and UND sample are closely related. Hence it is plausible to assume that the samples with an undetermined etiology could have resulted from small artery stroke.

### Good *vs* Poor Stroke Outcome

Principal component analysis of the stroke samples based on the clinical outcome (mRS) showed that all samples of good outcome (mRS<2; stroke, LA and CEmb) have been found to cluster along same panel with almost consistent distant between them. The poor outcome (mRS>2) stroke samples have been found to cluster away from the samples with good outcome (mRS<2; Figure 2b). The SA samples with good outcome (mRS<2) have been found to exhibit a unique pattern to that of LA and CEmb cases with good outcome (mRS<2). The reason for this observation is unknown. Similar observation has been made with hierachical clustering of the samples and miRNAs (Figure 2a).

## Discussion

Protein biomarkers from blood (serum/plasma) have been widely used over the years for clinical diagnosis and prognosis. Recently, circulating nucleic acids in peripheral blood samples have proven to be useful biomarkers in the diagnosis of stroke pathogenesis[15]. Cardioembolic and the large-vessel atherosclerotic stroke have been distinguished based on mRNA expression profiles[16]. miRNA-induced gene expression has also been shown to contribute extensively to the disease phenotype[17]. The apparent roles of miRNAs in diseases have led many researchers to probe into the molecular mechanisms underlying pathogenesis as well as to develop novel diagnostic and therapeutic agents[18]. We observed that the miRNAs are stably expressed and in circulation even after several months from the onset of stroke. This correlated with the observation by Chen et al[14] that miRNAs (in serum) are stable, resistant to nuclease digestion and almost consistent among individuals.

### MiRNA Expression in the Blood Samples of Stroke Patients

Distinct patterns of miRNAs indicative of the outcome of cerebral ischaemia have been observed. It is noteworthy that several miRNAs instead of a single miRNA, show changes during the progression of a disease. Another interesting observation is that more microRNAs are downregulated in all good outcome (mRS<2) stroke samples compared to normal controls, irrespective of subtype of stroke (Table S1).

miRNAs that are involved in endothelial function and angiogenesis (hsa-let-7f, miR-130a, -150, -17, -19a, -19b, -20a, -222 and -378)[18], vascular remodeling (miR-21, -126, -150), regulation of hematopoeisis (miR-223) as well as immune response (miR-20a, -17-92, -101, -150, -106a, -181a and -223)[7] have been found to be differentially regulated under ischemic conditions. Similarly, miRNAs that are expressed in hypoxic conditions (miR-23, -24, -26, -103, -107, -181)[19] have also been observed. Furthermore, the miRNAs that showed aberrant expression in cardiomyocyte hypertrophy and/or cardiac ischemia/reperfusion (miR-15, -16, -21, -23a, -29, -30a, -150 and -195)[20], [21] have also been detected in our profiling data.

The microRNAs, let-7a, let-7c, miR-16, -19b, -23a, -103, -106b, -185, -191, -320 and -451 that were present in both the blood and brain samples of rodent stroke (MCAo)[11] model have also been detected in the human blood samples used in this study. Interestingly, these groups of miRNAs are also known to participate in the regulation of angiogenesis (miR-320), hematopoetic process (miR-451), lymphocyte differentiation and proliferation (miR-103), cardiovascular function (miR-23a, -103, -451), metabolic processes (miR-451 and -320), immune response (miR-16) and hypoxic conditions (miR-23a, -103, -320). Microarray-based expression profiles in cancer cell lines revealed that a specific spectrum of microRNAs (including miR-23, -24, -26, -27, -103, -107, -181 and -210) has been induced in response to occlusion. Of these, miR-26, -107, and -210 decrease pro-apoptotic signaling in a hypoxic environment, thus suggesting a role in cell proliferation[19].

Upregulated miR-320 was associated with impaired angiogenesis in myocardial microvascular endothelial cells (MMVEC) of type 2 diabetic Goto-Kakizaki (GK) rats[22]. miR-320 has been observed to be marginally downregulated (fold change ∼1-1.5) in all stroke patients especially with good outcome (mRS<2). The downregulation of miR-320 could also lead to anti-apoptotic processes[23] that could be useful in restoration of normal cell or endovascular activities. Hence it could be predictive of a favorable outcome via activation of angiogenesis in stroke patients. However, the expression is relatively high (1.5–2 fold change) in CEmb, mRS>2. Similar observations were made in MCAo rat models[11].

Among the upregulated miRNAs in poor outcome (mRS>2) large artery and cardioembolic stroke, miR-103 and miR-29 have some significance. MiR-103 has been reported to regulate energy metabolism[6] while miR-29 regulates insulin resistance[24]. Thus, indicating their importance in energy utilization and production. Dysregulation of miR-29 has also been demonstrated in myocardial infarction[21].

We have also observed that subtypes of stroke could be predicted using the microRNA profiling. The 132 microRNAs listed in Table S2 could be useful in the prediction of the cause of stroke. These microRNAs show differential regulation (fold change) among the different subtypes. Sucharov et al[25] have shown that miR-125b is upregulated in idiopathic cardiomyopathic condition while the miR-150 is down regulated in both ischemic failing heart as well as in idiopathic cardiomyopathic conditions. Ischemic failing heart showed an upregulation for miR-197, miR-20a and miR-26b[25]. We observed that the expression of miR-150 was marginally higher, (fold change 1.16±0.03) for CEmb (mRS>2). However, the expression is lower in the mRS<2 conditions for both LA and CEmb samples (fold change 0.5±0.21 and 0.52±0.26 respectively). The expression of miR-26b was lowest in mRS<2 conditions for both LA and CEmb (fold change 0.19±0.23 and 0.28±0.20 respectively).

Further comparison of the individual sample profiles for C, E and F [LA1 (C) & LA2 (E); mRS = 2) and LA3 (F; mRS = 3); Table S5b] with the miRNA profiles of pooled samples miRNA profile showed that 72 miRNAs in the individual samples are specific to stroke (Table S3). Of these, 33 microRNAs were found to be expressed at either >2 or <0.5 fold change (bold font in Table 4) in the individual stroke samples. Among the highly upregulated microRNAs, the expression of miR-101, -106b, -130a, -144, -18a, -18b, -19a, -19b, -194, -22, -22, -29b, -29c and -363 were the highest for LA (mRS = 3) stroke sample and correlated to the profile observed for LA mRS>2. The expression value for hsa-let-7e, -149*, -484, -638, -652, -768-3p, and -923 were lowest in the LA (mRS = 3) sample. Interestingly, the expression of most of the let-7 family members (let-7b, -7c, -7d, -7f, -7g and -7i) were not altered (fold change ∼1.0) among all the large artery stroke samples.

**Table 4:** microRNAs and their target biological processes.

Process/Targets	microRNA/paralogs	
	Up regulated in this stroke study	Down regulated in this stroke study
Angiogenesis^18^	miR-19b, -130a, -15a,-16, -222, -320	
Hematopoietic regulation^5^	miR-16, -24, -30c, -106b, -223,	
Immune response^7^	miR-15a, -16, -24, -29a, -93, -181a, -223	
Lymphocyte regulation (differentiation & proliferation)^7^	miR-101, -181a, -16, -24, -22, -103, -30c, let-7c	
Cardiac/vascular function^20^	miR-23a, -23b, -24, -29a, -29c, -30a,-30c, -30d -103, -222	miR-126
Endothelial cell migration, differentiation and survival^18^	miR-126, -222	
Metabolic process regulation^6^	miR-130a, -29a, -29c, -320	
Hypoxia regulation^19^	miR-23a, -23b, -24, -103, -93, -181a, -15a, -16, -101, -126, -320, let-7c, let-7e,	miR-7, let-7a
Traumatic brain Injury (mouse brain)^12^	miR-103, -130a, -185, -191, -19b, -22, -222, -223, -23a, -23b, -30c, -320, -652, -744	
Stroke model (blood of rat-MCAo model)^11^	let-7c, miR-103, -106b, -16, -185, -191, -19b, -23a, 320,	let-7a
Stroke model (brain of rat MCAo model)^11,13^	let-7c, let-7d*, let-7e, miR-103, -126, -130a, -16, -181a, -185, -191, -222, -223, -23a, -23b, -24, -30c, -324-5p, -320,-29a, -29c, -7	let-7a

The biological processes that are affected by the microRNAs that are differentially expressed in the stroke samples have been analysed. List presented is based on published data.

### MiRNA Targets and Processes

Majority of the microRNAs that have been detected in the blood appear to be of cardiovascular/vascular, hematopoietic (hsa-let-7 family, miR-15, -16, -181a, -223)[5] or erythropoietic (miR-24, -221, -222, -320)[26] origin (Table 4). The differentially regulated miRs appear to be involved in angiogenic (pro-angiogenic: miR-130a, -378, -17–92 cluster, let-7f; anti-angiogenic: miR-15, -16, -20a, -20b, -222)[18], proliferative or vascular inflammatory functions (miR-21, -126) as well as hypoxic conditions (miR-23, -24, -26, -103, -107, -181)[19]. MicroRNAs that have been implicated in cardiovascular disease have also been found in the blood of our stroke patients. Key biological pathways that are affected by the differentially regulated microRNAs (Table S4) include MAPK signaling, TGFβ signaling, Wnt signaling and Focal Adhesion pathways. Presumably, these pathways are involved in important regulatory processes that lead to restoration and repair mechanisms.

Our analysis of the miRNA profiling has shown that the regulation of hypoxia, angiogenesis and erythropoiesis/hematopoiesis related processes are indeed the key events that occur during stroke recovery. We have shown that miRNAs could be used to differentiate cardioembolic, large artery and small artery strokes from each other. We have also shown that miRNA profiling could form an additional tool for the clinicians to determine the outcome of stroke.

## Materials and Methods

### Patient Selection and Blood Collection

#### Ethics Statement

This study has been approved by the Medical Ethics Committee of University of Malaya Medical Centre (UMMC) and the Institutional Review Board (IRB) of the National University of Singapore (NUS). Written consent was given by the patients for their information to be stored in the hospital database and used for research.

#### Clinical Methodology

Nineteen (Asian) stroke patients between the ages of 18 to 49 among those admitted via the neurology service at the University of Malaya Medical Centre (a major 900 bed teaching hospital serving a population of about 800,000) have been selected for the study. The study protocol included a standard neurological evaluation with subsequent review and follow up as out patients. In our study, we have utilized blood samples collected from stroke patients within 6–18 months in time scale from the index stroke. Ischaemic stroke was confirmed either with CT or MRI of the brain. Demographic data, medical history and conventional vascular risk factors were recorded in a standardized computerized database and abstracted from the medical records. Risk factors, if any, were defined in the following manner. Hypertension: BP above 140/90 mm Hg, Dyslipidemia: total cholesterol level of ≥6.7 mmol/l, triglyceride levels ≥1.8 mmol/l and HDL ≤1 mmol/l, Diabetes mellitus: elevated fasting blood glucose >6.1 mmol/l or HbA1c ≥7%. Smokers: who smoked ≥10 cigarettes per day for more than 1 year, Significant alcohol consumption: as ≥30 g of ethanol per day. Current medications (if any) were also recorded. Modified Rankin Score (mRS) [27] was evaluated at the time of blood sampling. Further diagnostic work-up included chest radiography, ECG, routine blood tests such as fasting lipid profile, fasting glucose and HbA1c. When routine stroke investigations were normal or negative, thrombophilia screen and detailed immunologic studies (anti-nuclear, anti-DNA and anti-ENA antibodies) were performed. Stroke events were classified when the patient was completely evaluated with the aetiology identified. Overall, the basis of the above classifications was based on clinical, imaging, routine and optional tests. Accordingly, the TOAST classification was applied. The stroke subjects used: a) Large-vessel atherosclerosis (n = 8); b) Small-vessel disease (n = 3); c) Cardioembolism (n = 5); d) Undetermined cause (n = 3). A total of 5 normal samples (n = 5) were also included as controls (Table S5a & S5b).

### Extraction of Micro RNAs from Blood Sample

Total RNA (+small RNA) was extracted from the blood samples using the Ribopure™ -Blood RNA isolation kit (Ambion, Austin, TX). The concentration of RNA was determined by a NanoDrop ND-1000 Spectrophotometer (Rockland, DE). The quality of RNAs was determined using denaturing gels (15% polyacrylamide and 1% agarose as needed)[11].

### MiRNA Stem-Loop Real-Time PCR

Quantitation of miRNAs was carried out using TaqMan Real-Time PCR[11]. Briefly, 10 ng of template RNA was reverse transcribed (in 15 µl) using stem-loop primer. For the PCR reaction, 1.33 µl (0.891 ng) of RT-product was used. PCR was carried out using the Applied Biosystems 7000 Sequence Detection System. Both RT- and PCR-reactions were performed in triplicate, in 3 separate experiments. miRNAs were considered as present when C_T_-values (threshold cycle) were lower than 30. The 18S rRNA was used as the housekeeping gene.

### μParaflo™ MicroRNA Microarray Assay and Analysis

Total RNA (2–5 µg) was size fractionated using a YM-100 Microcon (Millipore) and the small RNAs (<300 nt) isolated were 3′-extended using poly(A) polymerase. Test (pooled or individual stroke samples) and control samples were tagged at the poly(A) tails separately. Samples were pooled for all stroke (n = 19) [mRS<2 (n = 15), mRS>2 (n = 4)], large artery (LA) stroke (n = 8) [mRS<2 (n = 6), mRS>2 (n = 2)]; Cardioembolic (CEmb) stroke (n = 5) [mRS<2 (n = 3), mRS>2 (n = 2)]; Small artery (SA) stroke (mRS<2, n = 3) and stroke of undetermined cause (UND, mRS<2, n = 3). Hybridization was performed overnight on a μParaflo microfluidic chip using a micro-circulation pump (Atactic Technologies). On the chip, each detection probe consisted of a chemically modified nucleotide coding segment complementary to target microRNA (miRBase 10.1 and 11.0, http://microrna.sanger.ac.uk/sequences/). Hybridization images were scanned (GenePix 4000B, Molecular Device) and digitized using Array-Pro image analysis software (Media Cybernetics). Data were analyzed by first subtracting the background and then normalizing the signals using a LOWESS filter (Locally-weighted Regression;28]. The signal log ratio and p-values were calculated. Differentially detected signals were defined as those with less than 0.05 p-values. Microarray Analysis involved multiple sample analysis including background subtraction, t-Test/ANOVA analysis, clustering and principal component analysis. *t*-Test was performed between “control” and “test” sample groups and t-values were calculated for each miRNA. *p*-values were computed from the theoretical t-distribution. miRNAs with p-values ≤0.01 were selected for cluster analysis. The microarray data reported in this manuscript is described in accordance with MIAME guidelines. The clustering using hierarchical method was performed with average linkage and Euclidean distance metric. The clustering and principal component analysis (PCA) plot was generated using TIGR MeV (Multiple Experimental Viewer) software[29].

## Supporting Information

Table S1 miRNAs that have been detected in the peripheral blood of stroke patients. Fold Change (?SEM) of statistically significant (one way ANOVA, p value<0.05). The miRNAs that are upregulated in stroke (n = 19) is marked in red and the downregulated miRNAs are in green fonts.
(0.17 MB PDF)

Table S2 miRNA that showed mixed (up/and/or down) expression in stroke. Only fold change is shown (refer to Table S1 for ?SEM). The miRNAs that are upregulated in stroke (n = 19) is marked in red and the downregulated miRNAs are in green fonts.
(0.07 MB PDF)

Table S3 miRNA expression pattern of pooled and individual large artery stroke samples. Individual samples [LA1&2(mRS = 2) and LA(mRS = 3)] miRNA expression values is compared with the pooled sample miRNA profile of the larger artery stroke. The 72 miRNAs listed here correlated to the total miRNA that are differentially altered in the stroke sample. The microRNAs that are expressed in more than 2 fold change are in bold letters.
(0.04 MB XLS)

Table S4 miRNAs that are differentially expressed in stroke patients and the pathways that they possibly regulate. The miRNA:biological process relationship was predicted using miRNApath search tool (http://lgmb.fmrp.usp.br/mirnapath/tools.php and http://diana.cslab.ece.ntua.gr/) [30]. The pathways that have the largest number of genes affected by the miRNAs and with the highest [−ln(p-value)] are listed.
(0.07 MB DOC)

Table S5 Patient profile and medications (a) Demographic comparison and (b) Detailed medication and modified Rankin Score of young Asian stroke patients selected for the study. Controls (n = 5) included 3 males and 2 females.
(0.07 MB DOC)
