# Supplementary material for: Expression Profile of MicroRNAs in Young Stroke Patients
Source: PLoS One. 2009 Nov 2;4(11):e7689. doi: 10.1371/journal.pone.0007689 (PMC2765616; doi:10.1371/journal.pone.0007689)
Supplement: Table S1 — miRNAs that have been detected in the peripheral blood of stroke patients. Fold Change (?SEM) of statistically significant (one way ANOVA, p value<0.05). The miRNAs that are upregulated in stroke (n = 19) is marked in red and the downregulated miRNAs are in green fonts. (0.17 MB PDF) [file pone.0007689.s001.pdf]

anova p<0.05	miRNA	Stroke	*±SEM*	Stroke mRS<2	*±SEM*	Stroke mRS>2	*±SEM*	LA	*±SEM*	LA mRS<2	*±SEM*	LA mRS>2	*±SEM*	CEmb	*±SEM*	CEmb mRS<2	*±SEM*	CEmb mRS>2	*±SEM*	SA mRS<2	*±SEM*	UND mRS<2	*±SEM*
1.01E-17	**hsa-let-7a**	0.76	*0.00*	0.33	*0.00*	0.71	*0.01*	0.39	*0.05*	0.13	*0.00*	0.65	*0.05*	0.38	*0.07*	0.18	*0.05*	0.57	*0.11*	0.68	*0.05*	1.01	*0.08*
1.99E-12	**hsa-let-7b**	1.23	*0.02*	0.58	*0.02*	1.30	*0.03*	0.51	*0.09*	0.27	*0.01*	0.75	*0.12*	0.87	*0.16*	0.51	*0.01*	1.23	*0.23*	1.13	*0.01*	1.12	*0.03*
3.07E-12	**hsa-let-7c**	1.18	*0.00*	0.59	*0.01*	1.29	*0.02*	0.55	*0.09*	0.29	*0.02*	0.81	*0.13*	0.85	*0.02*	0.52	*0.01*	1.19	*0.04*	1.10	*0.06*	1.12	*0.07*
5.01E-13	**hsa-let-7d**	0.75	*0.03*	0.39	*0.00*	0.82	*0.05*	0.46	*0.14*	0.16	*0.03*	0.76	*0.16*	0.42	*0.03*	0.19	*0.01*	0.64	*0.03*	0.70	*0.00*	1.08	*0.18*
1.93E-08	**hsa-let-7d***	1.59	*0.00*	0.67	*0.03*	1.68	*0.01*	0.77	*0.06*	0.36	*0.04*	1.18	*0.09*	1.21	*0.02*	0.83	*0.01*	1.59	*0.03*	1.02	*0.01*	0.86	*0.03*
8.89E-15	**hsa-let-7e**	3.02	*0.11*	2.29	*0.00*	3.62	*0.09*	3.08	*0.02*	3.12	*0.04*	3.03	*0.09*	3.83	*0.00*	3.79	*0.05*	3.87	*0.04*	2.60	*0.05*	1.56	*0.00*
7.83E-18	**hsa-let-7f**	0.69	*0.05*	0.30	*0.06*	0.70	*0.07*	0.45	*0.08*	0.15	*0.07*	0.75	*0.08*	0.37	*0.09*	0.20	*0.09*	0.54	*0.10*	0.60	*0.08*	0.97	*0.07*
2.83E-16	**hsa-let-7g**	0.93	*0.04*	0.44	*0.01*	0.88	*0.02*	0.45	*0.06*	0.13	*0.01*	0.77	*0.07*	0.44	*0.04*	0.24	*0.01*	0.64	*0.06*	0.73	*0.01*	1.24	*0.04*
7.8E-15	**hsa-let-7i**	0.91	*0.04*	0.44	*0.06*	0.84	*0.03*	0.49	*0.05*	0.12	*0.10*	0.86	*0.08*	0.44	*0.02*	0.20	*0.09*	0.67	*0.05*	0.83	*0.04*	1.33	*0.05*
8.52E-12	**hsa-miR-101**	1.54	*0.13*	0.87	*0.10*	1.47	*0.06*	0.83	*0.02*	0.31	*0.13*	1.34	*0.01*	0.88	*0.16*	0.54	*0.08*	1.22	*0.20*	1.98	*0.07*	2.05	*0.13*
9.6E-12	**hsa-miR-103**	1.31	*0.00*	0.82	*0.01*	1.40	*0.04*	0.84	*0.11*	0.27	*0.05*	1.41	*0.13*	0.93	*0.05*	0.64	*0.01*	1.21	*0.07*	1.64	*0.03*	1.43	*0.18*
1.15E-13	**hsa-miR-106a**	1.49	*0.12*	1.02	*0.09*	1.51	*0.14*	0.84	*0.09*	0.22	*0.08*	1.45	*0.10*	0.84	*0.14*	0.50	*0.12*	1.18	*0.15*	1.77	*0.13*	1.82	*0.17*
2.07E-08	**hsa-miR-106b**	1.42	*0.14*	0.90	*0.16*	1.51	*0.13*	0.77	*0.16*	0.23	*0.12*	1.31	*0.17*	1.02	*0.08*	0.68	*0.10*	1.35	*0.06*	1.77	*0.15*	1.39	*0.24*
1.72E-12	**hsa-miR-106b***	1.42	*0.01*	0.73	*0.01*	1.52	*0.05*	0.80	*0.06*	0.36	*0.00*	1.25	*0.08*	1.03	*0.01*	0.75	*0.00*	1.31	*0.02*	1.37	*0.03*	1.30	*0.03*
1.03E-13	**hsa-miR-107**	1.17	*0.05*	0.64	*0.01*	1.22	*0.01*	0.76	*0.06*	0.22	*0.08*	1.30	*0.06*	0.72	*0.07*	0.51	*0.04*	0.92	*0.08*	1.37	*0.03*	1.29	*0.11*
1.93E-12	**hsa-miR-1184**	3.09	*0.04*	1.78	*0.08*	2.18	*0.02*	1.36	*0.03*	1.19	*0.08*	1.53	*0.02*	1.92	*0.06*	1.33	*0.05*	2.51	*0.11*	3.11	*0.01*	2.21	*0.06*
9.7E-09	**hsa-miR-1246**	1.66	*0.00*	1.44	*0.01*	1.68	*0.00*	1.78	*0.05*	2.25	*0.09*	1.29	*0.03*	2.09	*0.05*	2.07	*0.00*	2.10	*0.09*	1.29	*0.05*	1.67	*0.08*
7.53E-08	**hsa-miR-1255a**	1.03	*0.01*	0.68	*0.04*	1.25	*0.05*	0.73	*0.02*	0.58	*0.07*	0.87	*0.01*	0.90	*0.05*	0.59	*0.05*	1.21	*0.05*	0.95	*0.04*	1.11	*0.16*
0.000725	**hsa-miR-1259**	0.71	*0.06*	0.17	*0.18*	0.92	*0.05*	0.74	*0.08*	0.62	*0.07*	0.85	*0.19*	0.54	*0.20*	0.22	*0.92*	0.78	*0.02*	0.34	*0.03*	0.63	*0.01*
1.08E-20	**hsa-miR-125b-1***	1.98	*0.00*	1.29	*0.02*	2.58	*0.01*	1.15	*0.05*	0.96	*0.01*	1.33	*0.07*	2.07	*0.01*	1.00	*0.05*	3.14	*0.00*	1.90	*0.00*	1.70	*0.02*
4.78E-11	**hsa-miR-126**	0.79	*0.02*	0.31	*0.01*	0.75	*0.05*	0.50	*0.07*	0.16	*0.02*	0.84	*0.08*	0.37	*0.03*	0.20	*0.01*	0.53	*0.05*	0.71	*0.05*	0.92	*0.14*
1.46E-11	**hsa-miR-1261**	3.95	*0.07*	1.81	*0.04*	5.52	*0.05*	1.67	*0.06*	1.51	*0.06*	1.83	*0.06*	4.02	*0.02*	1.35	*0.21*	6.65	*0.07*	2.87	*0.07*	3.08	*0.08*
7.8E-24	**hsa-miR-1264**	1.53	*0.05*	1.03	*0.03*	1.87	*0.07*	1.03	*0.02*	0.32	*0.05*	1.74	*0.03*	1.21	*0.01*	0.76	*0.03*	1.65	*0.01*	1.64	*0.00*	1.49	*0.02*
1.14E-11	**hsa-miR-1265**	1.36	*0.07*	0.75	*0.06*	1.20	*0.08*	0.99	*0.06*	0.70	*0.03*	1.28	*0.08*	1.00	*0.09*	0.80	*0.05*	1.20	*0.13*	1.13	*0.04*	1.18	*0.07*
1.05E-15	**hsa-miR-1275**	3.06	*0.04*	2.27	*0.02*	3.01	*0.02*	1.44	*0.05*	1.63	*0.03*	1.24	*0.07*	2.71	*0.06*	2.51	*0.04*	2.91	*0.08*	2.32	*0.04*	3.36	*0.03*
2.75E-13	**hsa-miR-1280**	1.99	*0.03*	1.67	*0.04*	2.14	*0.00*	1.10	*0.05*	0.96	*0.01*	1.24	*0.08*	1.72	*0.05*	1.47	*0.02*	1.98	*0.07*	2.22	*0.01*	2.09	*0.04*
3.56E-09	**hsa-miR-1284**	1.01	*0.02*	0.59	*0.13*	1.07	*0.06*	0.66	*0.01*	0.48	*0.05*	0.84	*0.04*	0.66	*0.08*	0.37	*0.24*	0.95	*0.03*	0.84	*0.05*	0.94	*0.03*
5.14E-10	**hsa-miR-1285**	1.87	*0.06*	1.22	*0.06*	1.68	*0.07*	1.34	*0.01*	1.54	*0.00*	1.14	*0.03*	1.37	*0.02*	1.04	*0.02*	1.70	*0.03*	1.30	*0.04*	1.39	*0.01*
6.98E-13	**hsa-miR-1290**	2.01	*0.03*	1.70	*0.04*	1.98	*0.02*	2.14	*0.01*	2.71	*0.01*	1.57	*0.05*	2.27	*0.05*	2.20	*0.05*	2.33	*0.05*	1.37	*0.01*	2.27	*0.01*
2.97E-18	**hsa-miR-129-5p**	1.73	*0.13*	1.13	*0.11*	2.07	*0.11*	1.00	*0.14*	0.93	*0.13*	1.07	*0.14*	1.95	*0.12*	1.00	*0.16*	2.89	*0.11*	1.52	*0.13*	1.54	*0.10*
3.09E-15	**hsa-miR-1308**	1.03	*0.02*	0.57	*0.02*	0.98	*0.03*	0.90	*0.05*	0.77	*0.02*	1.03	*0.07*	0.75	*0.03*	0.64	*0.03*	0.86	*0.03*	0.63	*0.02*	0.67	*0.02*
4.13E-10	**hsa-miR-130a**	1.56	*0.06*	0.80	*0.09*	1.64	*0.10*	0.99	*0.09*	0.30	*0.09*	1.69	*0.09*	0.92	*0.00*	0.62	*0.04*	1.21	*0.03*	1.70	*0.12*	1.35	*0.04*
3.37E-12	**hsa-miR-130b**	1.78	*0.07*	1.17	*0.04*	1.90	*0.05*	0.89	*0.00*	0.30	*0.10*	1.47	*0.02*	1.35	*0.04*	1.06	*0.04*	1.65	*0.04*	2.33	*0.07*	1.84	*0.03*
7.06E-14	**hsa-miR-138-1***	1.20	*0.02*	0.94	*0.01*	1.20	*0.06*	0.93	*0.01*	0.35	*0.00*	1.51	*0.01*	0.98	*0.09*	0.66	*0.00*	1.29	*0.13*	1.43	*0.05*	1.20	*0.04*
8.23E-08	**hsa-miR-140-3p**	1.29	*0.02*	0.76	*0.06*	1.20	*0.03*	0.72	*0.02*	0.29	*0.01*	1.15	*0.03*	0.89	*0.04*	0.73	*0.06*	1.05	*0.11*	1.12	*0.04*	0.99	*0.02*
1.74E-17	**hsa-miR-142-3p**	0.59	*0.02*	0.28	*0.01*	0.69	*0.01*	0.43	*0.08*	0.18	*0.03*	0.68	*0.10*	0.37	*0.03*	0.15	*0.03*	0.60	*0.05*	0.70	*0.01*	0.86	*0.06*
1.4E-10	**hsa-miR-142-5p**	1.42	*0.02*	0.86	*0.02*	1.32	*0.02*	0.80	*0.13*	0.27	*0.02*	1.32	*0.15*	0.85	*0.10*	0.55	*0.01*	1.14	*0.15*	1.24	*0.01*	1.54	*0.01*
3.74E-13	**hsa-miR-144**	1.35	*0.08*	0.87	*0.07*	1.61	*0.07*	0.85	*0.02*	0.42	*0.06*	1.28	*0.01*	1.17	*0.05*	0.65	*0.09*	1.70	*0.03*	1.70	*0.03*	1.57	*0.00*

1.73E-16	**hsa-miR-144***	0.69	0.06	1.30	0.04	0.71	0.05	0.60	0.06	0.06	0.02	1.14	0.06	0.32	0.09	0.05	0.01	0.59	0.10	1.07	0.05	1.43	0.07
3.15E-07	**hsa-miR-148b**	1.24	0.04	0.70	0.01	1.24	0.04	0.56	0.17	0.16	0.01	0.95	0.20	0.77	0.05	0.50	0.03	1.04	0.09	0.91	0.03	1.16	0.18
1.37E-12	**hsa-miR-149***	1.68	0.11	1.01	0.08	2.25	0.10	1.18	0.09	0.78	0.10	1.58	0.08	1.94	0.11	0.99	0.10	2.89	0.11	1.21	0.08	1.38	0.08
3.85E-07	**hsa-miR-150**	2.22	0.34	0.69	0.21	1.62	0.20	0.61	0.10	0.50	0.21	0.72	0.02	0.84	0.10	0.52	0.26	1.16	0.03	1.01	0.29	1.62	0.09
7.67E-07	**hsa-miR-151-3p**	2.38	0.10	1.34	0.13	2.48	0.06	1.00	0.28	0.29	0.22	1.70	0.29	1.71	0.09	1.08	0.10	2.34	0.08	2.65	0.09	2.40	0.08
1.62E-06	**hsa-miR-151-5p**	1.47	0.29	0.84	0.32	1.33	0.14	0.75	0.15	0.12	0.49	1.38	0.12	0.66	0.17	0.28	0.28	1.04	0.14	1.56	0.13	1.78	0.13
3.05E-15	**hsa-miR-15a**	1.07	0.01	0.57	0.04	1.18	0.01	0.63	0.07	0.24	0.00	1.03	0.09	0.66	0.03	0.36	0.01	0.96	0.04	1.21	0.05	1.29	0.03
9.47E-07	**hsa-miR-15b**	0.94	0.01	0.53	0.05	0.93	0.07	0.59	0.04	0.48	0.09	0.70	0.00	0.70	0.08	0.59	0.06	0.81	0.09	0.65	0.06	0.66	0.11
3.4E-14	**hsa-miR-16**	1.02	0.02	0.62	0.08	1.03	0.03	0.67	0.02	0.30	0.03	1.03	0.03	0.74	0.02	0.46	0.03	1.02	0.04	1.03	0.03	1.01	0.04
1.07E-07	**hsa-miR-16-2***	1.42	0.09	0.70	0.12	1.82	0.03	0.84	0.19	0.09	0.12	1.60	0.20	1.01	0.08	0.38	0.49	1.61	0.01	1.38	0.09	1.82	0.06
1.19E-22	**hsa-miR-17**	1.26	0.01	0.79	0.00	1.26	0.00	0.72	0.05	0.19	0.01	1.26	0.06	0.67	0.03	0.41	0.00	0.94	0.05	1.48	0.01	1.47	0.01
1.28E-05	**hsa-miR-17***	2.02	0.01	0.84	0.03	1.80	0.03	0.73	0.12	0.29	0.01	1.15	0.15	1.41	0.08	0.94	0.01	1.88	0.13	1.63	0.00	1.70	0.11
1.46E-08	**hsa-miR-181a**	1.64	0.04	1.56	0.06	1.74	0.06	1.14	0.04	1.06	0.02	1.22	0.05	1.89	0.03	1.64	0.02	2.15	0.04	1.76	0.03	2.26	0.06
4.69E-08	**hsa-miR-182**	1.35	0.10	0.59	0.12	1.13	0.13	0.48	0.10	0.09	0.05	0.86	0.11	0.68	0.18	0.46	0.09	0.89	0.22	1.37	0.12	1.39	0.19
2.15E-09	**hsa-miR-1826**	1.01	0.04	0.61	0.09	0.97	0.03	0.91	0.03	0.89	0.02	0.94	0.04	0.74	0.03	0.59	0.02	0.89	0.06	0.71	0.04	0.88	0.01
0.000408	**hsa-miR-1827**	1.24	0.09	0.72	0.13	1.15	0.03	0.85	0.17	0.76	0.11	0.94	0.22	1.03	0.15	0.91	0.11	1.15	0.19	0.82	0.13	1.23	0.13
3.24E-15	**hsa-miR-183**	1.32	0.01	0.78	0.01	1.14	0.01	0.54	0.04	0.23	0.10	0.84	0.07	0.99	0.03	0.83	0.06	1.15	0.10	1.22	0.01	1.10	0.01
2.14E-09	**hsa-miR-183***	1.88	0.02	0.96	0.04	2.27	0.01	1.02	0.04	0.84	0.01	1.20	0.07	1.83	0.04	0.76	0.03	2.89	0.06	1.59	0.00	1.34	0.14
7.41E-14	**hsa-miR-184**	1.93	0.04	1.75	0.08	2.82	0.05	1.33	0.09	0.99	0.09	1.67	0.09	2.29	0.10	1.45	0.13	3.14	0.09	2.43	0.08	2.16	0.04
6.3E-08	**hsa-miR-185**	1.78	0.03	0.91	0.06	1.90	0.03	0.93	0.04	0.25	0.03	1.62	0.06	1.10	0.15	0.92	0.05	1.28	0.23	1.54	0.02	1.39	0.06
6.02E-19	**hsa-miR-185***	2.15	0.05	1.15	0.04	2.93	0.02	1.03	0.07	0.82	0.04	1.25	0.09	2.32	0.01	0.95	0.03	3.70	0.00	1.42	0.01	1.75	0.01
3.18E-09	**hsa-miR-186**	0.84	0.03	0.48	0.03	0.81	0.01	0.44	0.03	0.21	0.06	0.68	0.02	0.67	0.07	0.52	0.00	0.81	0.11	0.83	0.04	0.74	0.12
5.67E-12	**hsa-miR-18a**	1.96	0.07	1.22	0.11	2.13	0.12	1.09	0.27	0.24	0.09	1.93	0.29	1.00	0.16	0.68	0.11	1.32	0.18	2.74	0.12	2.36	0.16
9.16E-16	**hsa-miR-18b**	1.46	0.04	1.06	0.04	1.59	0.00	0.87	0.11	0.21	0.03	1.53	0.12	0.81	0.08	0.51	0.00	1.11	0.12	1.90	0.05	1.86	0.04
1.92E-07	**hsa-miR-191**	1.47	0.13	0.92	0.14	1.47	0.11	0.70	0.01	0.32	0.14	1.09	0.03	1.06	0.17	0.87	0.15	1.25	0.19	1.34	0.12	1.33	0.16
4.7E-13	**hsa-miR-194**	1.79	0.00	0.84	0.08	1.93	0.02	0.87	0.17	0.20	0.05	1.53	0.20	1.05	0.02	0.76	0.04	1.34	0.06	1.64	0.01	1.39	0.02
6.9E-12	**hsa-miR-195**	1.06	0.06	0.27	0.16	1.01	0.04	0.45	0.11	0.08	0.17	0.82	0.10	0.45	0.02	0.16	0.17	0.73	0.06	1.32	0.06	1.24	0.17
4.61E-19	**hsa-miR-19a**	1.41	0.03	0.81	0.06	1.33	0.03	0.82	0.01	0.30	0.00	1.35	0.02	0.87	0.03	0.51	0.03	1.22	0.03	1.23	0.01	1.49	0.07
1.21E-12	**hsa-miR-19b**	1.65	0.10	1.10	0.06	1.47	0.04	0.88	0.14	0.52	0.06	1.24	0.17	1.18	0.11	0.86	0.08	1.49	0.12	1.62	0.02	1.74	0.19
2.42E-13	**hsa-miR-20a**	0.95	0.10	0.50	0.22	0.85	0.20	0.45	0.18	0.18	0.09	0.73	0.20	0.40	0.12	0.21	0.24	0.59	0.08	0.74	0.15	1.04	0.04
9.52E-12	**hsa-miR-20b**	1.29	0.09	0.71	0.06	1.29	0.06	0.69	0.14	0.12	0.04	1.25	0.15	0.65	0.09	0.36	0.04	0.93	0.11	1.37	0.03	1.46	0.14
3.83E-10	**hsa-miR-21**	1.34	0.14	0.58	0.15	1.28	0.15	0.75	0.00	0.17	0.17	1.33	0.02	0.75	0.12	0.35	0.12	1.15	0.12	1.72	0.15	1.79	0.20
4.83E-09	**hsa-miR-22**	1.26	0.01	0.85	0.05	1.28	0.02	0.59	0.05	0.30	0.02	0.87	0.06	1.17	0.02	1.03	0.01	1.30	0.04	0.99	0.07	1.00	0.12
8.16E-11	**hsa-miR-222**	1.31	0.10	0.85	0.08	1.26	0.10	0.67	0.06	0.17	0.04	1.18	0.06	0.83	0.09	0.56	0.07	1.11	0.10	1.70	0.10	1.57	0.11
2.51E-12	**hsa-miR-223**	1.44	0.04	0.88	0.08	1.42	0.02	0.93	0.06	0.25	0.01	1.61	0.08	0.57	0.03	0.35	0.06	0.80	0.02	1.71	0.04	1.72	0.11
6.33E-12	**hsa-miR-23a**	1.63	0.00	1.21	0.01	1.37	0.03	0.86	0.02	0.37	0.03	1.36	0.02	0.80	0.12	0.61	0.30	0.97	0.01	1.78	0.11	1.94	0.06
3.04E-16	**hsa-miR-23b**	1.37	0.05	0.97	0.03	1.18	0.04	0.67	0.09	0.21	0.42	1.10	0.20	0.78	0.10	0.64	0.06	0.91	0.13	1.39	0.06	1.75	0.10
1.6E-11	**hsa-miR-24**	1.23	0.10	0.87	0.11	1.06	0.04	0.58	0.16	0.25	0.04	0.91	0.20	0.71	0.01	0.60	0.04	0.82	0.01	1.17	0.05	1.50	0.01
1.35E-08	**hsa-miR-24-1***	1.19	0.03	0.97	0.04	1.30	0.02	0.81	0.00	0.39	0.00	1.24	0.00	1.27	0.02	0.83	0.01	1.72	0.03	1.24	0.10	1.27	0.03
2.56E-15	**hsa-miR-25**	1.24	0.06	0.80	0.02	1.21	0.04	0.56	0.10	0.27	0.02	0.85	0.14	0.80	0.05	0.52	0.03	1.08	0.05	1.23	0.07	1.44	0.04
2.54E-08	**hsa-miR-25***	1.64	0.07	1.21	0.04	1.91	0.03	1.48	0.09	1.27	0.07	1.66	0.21	2.18	0.20	1.26	0.04	3.07	0.26	1.62	0.05	1.45	0.17
2.96E-15	**hsa-miR-26a**	1.17	0.00	0.70	0.05	1.10	0.02	0.58	0.08	0.18	0.04	0.98	0.10	0.67	0.00	0.43	0.01	0.91	0.00	1.28	0.07	1.29	0.00

9.65E-11	hsa-miR-26b	0.97	0.21	0.47	0.20	0.92	0.20	0.57	0.00	0.19	0.23	0.94	0.05	0.48	0.18	0.28	0.20	0.68	0.17	1.00	0.20	1.19	0.11
5.41E-17	hsa-miR-299-3p	2.15	0.03	1.45	0.03	1.79	0.01	1.16	0.00	1.41	0.01	0.90	0.02	1.84	0.00	1.39	0.01	2.29	0.00	1.71	0.03	1.27	0.08
4.16E-14	hsa-miR-29a	1.40	0.03	0.85	0.02	1.48	0.04	0.75	0.02	0.28	0.09	1.22	0.05	0.96	0.02	0.64	0.03	1.29	0.01	1.34	0.06	1.42	0.02
1.69E-09	hsa-miR-29b	1.58	0.04	1.10	0.12	1.45	0.07	0.84	0.02	0.44	0.06	1.23	0.01	0.99	0.07	0.41	0.04	1.56	0.10	2.03	0.02	1.77	0.17
5.67E-10	hsa-miR-29c	2.34	0.01	1.21	0.12	2.38	0.01	1.08	0.09	0.30	0.00	1.85	0.10	1.58	0.03	1.01	0.25	2.13	0.08	3.14	0.04	2.25	0.02
3.64E-08	hsa-miR-300	1.28	0.01	0.84	0.01	1.11	0.01	0.56	0.02	0.56	0.02	0.56	0.05	1.10	0.01	0.78	0.01	1.42	0.00	1.31	0.02	1.29	0.04
4.62E-09	hsa-miR-301a	1.56	0.05	0.88	0.12	1.63	0.05	0.97	0.10	0.20	0.14	1.74	0.13	0.81	0.07	0.47	0.13	1.15	0.04	2.05	0.10	2.07	0.03
9.64E-09	hsa-miR-30a	1.30	0.05	0.86	0.07	1.41	0.04	0.72	0.09	0.34	0.04	1.11	0.10	1.05	0.02	0.72	0.06	1.38	0.00	1.33	0.04	1.34	0.02
4.9E-10	hsa-miR-30b	0.98	0.03	0.57	0.02	1.00	0.04	0.59	0.01	0.16	0.02	1.01	0.01	0.53	0.06	0.28	0.02	0.78	0.07	1.07	0.00	1.24	0.07
1.77E-14	hsa-miR-30c	1.29	0.10	0.73	0.11	1.17	0.06	0.66	0.04	0.17	0.10	1.16	0.03	0.64	0.09	0.39	0.06	0.89	0.10	1.30	0.08	1.53	0.03
3.68E-09	hsa-miR-30d	1.48	0.05	0.99	0.03	1.66	0.07	0.77	0.03	0.39	0.07	1.16	0.06	1.36	0.08	1.03	0.05	1.68	0.10	1.29	0.07	1.22	0.09
3.59E-11	hsa-miR-30e	1.16	0.00	0.74	0.05	1.18	0.02	0.71	0.05	0.22	0.01	1.20	0.06	0.73	0.06	0.40	0.02	1.05	0.09	1.21	0.01	1.47	0.04
1.29E-08	hsa-miR-32*	1.04	0.01	0.48	0.01	1.15	0.04	0.66	0.04	0.54	0.03	0.78	0.08	0.75	0.02	0.56	0.00	0.95	0.04	0.77	0.00	0.76	0.03
2.62E-12	hsa-miR-320a	2.07	0.11	1.19	0.10	2.60	0.10	1.10	0.01	0.70	0.11	1.50	0.03	2.18	0.05	1.57	0.09	2.80	0.03	1.75	0.12	1.35	0.11
1.25E-08	hsa-miR-320b	1.52	0.03	0.88	0.01	1.81	0.00	0.86	0.07	0.57	0.02	1.15	0.09	1.53	0.05	1.19	0.05	1.87	0.04	1.26	0.04	1.03	0.05
3.06E-10	hsa-miR-320c	1.39	0.09	0.73	0.00	1.77	0.00	0.78	0.08	0.47	0.02	1.09	0.11	1.40	0.01	1.00	0.02	1.79	0.01	1.04	0.02	0.84	0.08
3.4E-09	hsa-miR-320d	1.56	0.07	0.88	0.14	1.96	0.11	0.87	0.01	0.60	0.07	1.13	0.05	1.55	0.08	1.14	0.12	1.97	0.06	1.28	0.09	1.04	0.00
1.27E-08	hsa-miR-324-5p	1.26	0.18	0.43	0.25	1.14	0.20	0.44	0.35	0.15	0.35	0.73	0.35	0.65	0.13	0.46	0.20	0.83	0.09	0.84	0.13	0.70	0.23
2.18E-07	hsa-miR-326	1.31	0.13	0.96	0.10	1.24	0.14	1.00	0.19	0.68	0.22	1.31	0.17	0.74	0.13	0.49	0.16	0.99	0.11	1.63	0.16	1.33	0.04
1.39E-06	hsa-miR-331-3p	1.44	0.05	0.72	0.07	1.71	0.01	0.89	0.20	0.41	0.03	1.37	0.25	1.27	0.10	0.94	0.05	1.60	0.13	1.15	0.03	0.95	0.12
1.6E-07	hsa-miR-339-5p	2.26	0.28	1.40	0.28	1.86	0.29	0.98	0.47	0.48	0.27	1.48	0.53	1.74	0.28	1.78	0.27	1.71	0.29	2.08	0.26	1.75	0.28
5.81E-08	hsa-miR-342-3p	1.29	0.01	0.66	0.22	1.13	0.08	0.65	0.04	0.15	0.16	1.15	0.06	0.56	0.02	0.36	0.08	0.75	0.01	0.83	0.04	1.39	0.02
6.56E-11	hsa-miR-34b	1.20	0.06	0.88	0.10	1.38	0.05	1.03	0.08	0.99	0.00	1.06	0.16	1.14	0.01	0.53	0.07	1.75	0.01	1.08	0.02	1.15	0.03
8.91E-10	hsa-miR-361-5p	1.58	0.03	1.06	0.05	1.79	0.05	0.88	0.14	0.23	0.05	1.52	0.17	1.23	0.02	0.88	0.23	1.54	0.17	1.80	0.01	2.05	0.16
7.01E-08	hsa-miR-362-5p	1.78	0.00	0.78	0.10	1.85	0.02	0.75	0.16	0.18	0.08	1.31	0.19	1.12	0.11	0.55	0.14	1.67	0.19	1.73	0.02	1.54	0.03
8.88E-09	hsa-miR-363	1.24	0.18	0.57	0.20	1.42	0.18	0.67	0.02	0.24	0.20	1.09	0.07	0.95	0.15	0.57	0.21	1.33	0.12	1.10	0.22	1.10	0.11
1.39E-14	hsa-miR-374a	0.93	0.01	0.50	0.06	0.96	0.02	0.66	0.11	0.16	0.04	1.16	0.13	0.47	0.02	0.25	0.07	0.68	0.05	1.00	0.03	1.11	0.04
5.26E-12	hsa-miR-378	1.43	0.03	0.82	0.01	1.30	0.04	0.63	0.01	0.25	0.08	1.01	0.00	1.14	0.02	0.96	0.01	1.31	0.05	1.22	0.03	1.23	0.08
1.82E-09	hsa-miR-423-3p	1.35	0.01	0.77	0.03	1.44	0.04	0.76	0.05	0.44	0.02	1.07	0.06	1.21	0.01	1.11	0.01	1.32	0.02	0.96	0.00	0.90	0.00
2.61E-07	hsa-miR-423-5p	1.81	0.11	0.86	0.11	2.22	0.12	0.91	0.01	0.68	0.08	1.14	0.03	1.57	0.11	1.18	0.11	1.96	0.11	1.11	0.11	0.92	0.14
1.51E-08	hsa-miR-425	1.29	0.04	0.91	0.02	1.41	0.03	0.82	0.05	0.44	0.03	1.19	0.06	1.15	0.04	0.80	0.02	1.50	0.05	1.19	0.01	1.45	0.03
0.04609	hsa-miR-451	1.00	0.00	1.00	0.00	1.00	0.00	1.00	0.00	1.00	0.00	1.00	0.00	1.00	0.00	1.00	0.00	1.00	0.00	1.00	0.00	1.00	0.00
1.24E-08	hsa-miR-484	1.49	0.02	0.86	0.05	1.61	0.09	0.82	0.06	0.53	0.06	1.11	0.06	1.32	0.12	1.03	0.07	1.61	0.14	0.99	0.07	0.90	0.10
0.00014	hsa-miR-485-3p	1.53	0.06	0.78	0.04	1.74	0.06	0.98	0.04	0.34	0.05	1.62	0.03	1.43	0.07	0.78	0.03	2.08	0.10	1.00	0.15	1.65	0.03
0.001086	hsa-miR-486-5p	1.21	0.02	1.19	0.00	1.21	0.02	1.06	0.00	0.90	0.02	1.21	0.02	1.21	0.02	1.21	0.02	1.21	0.02	1.08	0.02	1.19	0.03
9.54E-08	hsa-miR-487b	1.27	0.07	0.79	0.03	1.32	0.05	0.76	0.18	0.17	0.16	1.34	0.22	0.99	0.04	0.64	0.02	1.34	0.05	1.62	0.04	0.90	0.02
3.36E-05	hsa-miR-491-3p	1.57	0.12	1.25	0.10	1.58	0.11	1.01	0.04	0.80	0.12	1.23	0.01	1.47	0.11	1.40	0.11	1.54	0.11	1.37	0.11	1.16	0.17
1.36E-11	hsa-miR-494	2.07	0.01	1.53	0.01	2.03	0.01	1.47	0.04	1.42	0.02	1.51	0.11	1.92	0.05	1.60	0.03	2.24	0.06	1.76	0.03	1.97	0.03
3.35E-08	hsa-miR-500	2.07	0.10	1.50	0.11	2.72	0.11	1.41	0.10	0.31	0.10	2.51	0.12	1.81	0.11	1.24	0.12	2.39	0.10	2.81	0.13	3.41	0.22
7.05E-08	hsa-miR-501-5p	1.52	0.05	0.65	0.08	1.69	0.04	0.74	0.05	0.30	0.11	1.17	0.04	1.27	0.10	0.76	0.03	1.77	0.13	1.20	0.06	0.70	0.33
1.19E-11	hsa-miR-513a-5p	2.04	0.00	1.30	0.03	2.01	0.02	1.29	0.05	1.30	0.00	1.28	0.09	1.89	0.01	1.62	0.00	2.16	0.01	1.58	0.00	1.76	0.01
1.19E-18	hsa-miR-519d	1.78	0.01	1.29	0.05	2.10	0.00	1.22	0.01	0.36	0.08	2.09	0.03	1.29	0.02	1.03	0.01	1.56	0.03	2.21	0.04	1.98	0.04

9.13E-06	**hsa-miR-519e**	0.63	*0.05*	0.59	*0.00*	0.90	*0.02*	0.78	*0.16*	0.58	*0.06*	0.97	*0.21*	0.43	*0.03*	0.30	*0.07*	0.57	*0.00*	0.93	*0.06*	0.76	*0.08*
4.23E-05	**hsa-miR-519e***	2.76	*0.05*	2.04	*0.06*	3.53	*0.08*	2.18	*0.12*	1.25	*0.06*	3.11	*0.14*	2.77	*0.12*	1.75	*0.08*	3.79	*0.14*	3.23	*0.07*	2.86	*0.17*
0.000314	**hsa-miR-532-5p**	1.65	*0.12*	0.83	*0.10*	1.74	*0.11*	0.66	*0.13*	0.29	*0.12*	1.02	*0.14*	1.07	*0.29*	0.54	*0.23*	1.59	*0.31*	2.10	*0.12*	2.07	*0.24*
5.2E-13	**hsa-miR-549**	1.65	*0.06*	1.26	*0.03*	1.72	*0.04*	1.21	*0.11*	1.23	*0.05*	1.19	*0.16*	1.62	*0.05*	0.99	*0.03*	2.25	*0.06*	1.74	*0.02*	1.52	*0.08*
2.43E-08	**hsa-miR-550**	1.81	*0.09*	1.06	*0.10*	1.91	*0.09*	1.32	*0.04*	1.29	*0.12*	1.33	*0.04*	1.52	*0.12*	1.09	*0.12*	1.95	*0.13*	1.49	*0.10*	1.36	*0.15*
3.13E-05	**hsa-miR-550***	1.37	*0.15*	0.94	*0.13*	1.50	*0.07*	0.85	*0.06*	0.41	*0.04*	1.29	*0.09*	1.26	*0.05*	1.04	*0.12*	1.47	*0.00*	1.27	*0.11*	1.25	*0.09*
4.77E-07	**hsa-miR-551b**	1.12	*0.07*	0.85	*0.01*	1.13	*0.04*	0.89	*0.01*	0.89	*0.05*	0.89	*0.02*	0.84	*0.01*	0.66	*0.02*	1.02	*0.00*	1.05	*0.03*	1.25	*0.13*
2.04E-07	**hsa-miR-574-5p**	1.05	*0.11*	0.38	*0.17*	1.32	*0.22*	0.56	*0.15*	0.50	*0.12*	0.62	*0.18*	0.74	*0.06*	0.43	*0.22*	1.04	*0.00*	0.74	*0.15*	0.63	*0.18*
7.16E-10	**hsa-miR-584**	1.87	*0.11*	1.20	*0.09*	2.03	*0.11*	1.16	*0.10*	0.70	*0.10*	1.62	*0.10*	1.48	*0.10*	0.95	*0.09*	2.02	*0.11*	2.39	*0.10*	1.80	*0.14*
1.82E-09	**hsa-miR-602**	2.48	*0.03*	1.83	*0.08*	3.06	*0.03*	1.47	*0.01*	1.37	*0.08*	1.56	*0.10*	2.57	*0.06*	1.94	*0.02*	3.19	*0.08*	2.45	*0.10*	2.46	*0.04*
2.65E-14	**hsa-miR-628-3p**	1.67	*0.11*	0.94	*0.13*	2.30	*0.07*	0.96	*0.10*	0.87	*0.05*	1.04	*0.15*	2.09	*0.03*	0.77	*0.07*	3.41	*0.02*	1.33	*0.08*	1.23	*0.05*
1.35E-10	**hsa-miR-634**	1.70	*0.07*	1.49	*0.10*	2.31	*0.05*	0.94	*0.18*	0.57	*0.11*	1.31	*0.21*	1.41	*0.00*	1.00	*0.05*	1.81	*0.03*	2.72	*0.03*	1.87	*0.15*
1.89E-14	**hsa-miR-637**	2.25	*0.01*	1.30	*0.04*	2.96	*0.04*	1.13	*0.04*	1.10	*0.00*	1.16	*0.08*	2.43	*0.06*	0.95	*0.01*	3.92	*0.08*	2.00	*0.02*	1.87	*0.10*
7.75E-08	**hsa-miR-638**	1.52	*0.15*	1.16	*0.14*	2.36	*0.19*	1.10	*0.25*	1.09	*0.15*	1.10	*0.34*	2.23	*0.20*	0.92	*0.10*	3.54	*0.23*	1.30	*0.15*	1.62	*0.33*
1.79E-05	**hsa-miR-642**	0.83	*0.17*	0.57	*0.14*	0.83	*0.15*	0.60	*0.09*	0.48	*0.18*	0.71	*0.02*	0.83	*0.22*	0.75	*0.29*	0.91	*0.16*	0.70	*0.13*	0.86	*0.09*
4.74E-08	**hsa-miR-652**	1.14	*0.07*	0.66	*0.06*	1.30	*0.07*	0.71	*0.16*	0.43	*0.07*	0.99	*0.20*	1.03	*0.06*	0.72	*0.08*	1.33	*0.05*	1.01	*0.06*	0.88	*0.07*
3.56E-09	**hsa-miR-665**	3.02	*0.10*	1.81	*0.10*	3.26	*0.10*	2.33	*0.03*	2.26	*0.07*	2.39	*0.11*	2.67	*0.02*	2.39	*0.04*	2.96	*0.01*	2.05	*0.06*	1.57	*0.05*
9.95E-05	**hsa-miR-668**	1.96	*0.06*	0.97	*0.04*	2.09	*0.00*	1.25	*0.09*	1.05	*0.03*	1.44	*0.12*	1.33	*0.01*	0.70	*0.04*	1.97	*0.03*	1.91	*0.03*	1.70	*0.29*
1.28E-10	**hsa-miR-7**	0.81	*0.08*	0.34	*0.04*	0.86	*0.05*	0.48	*0.13*	0.25	*0.24*	0.72	*0.09*	0.47	*0.04*	0.23	*0.05*	0.71	*0.04*	0.93	*0.02*	1.58	*0.07*
1.86E-05	**hsa-miR-720**	1.63	*0.15*	0.91	*0.11*	1.51	*0.12*	0.96	*0.02*	0.65	*0.15*	1.27	*0.04*	1.15	*0.18*	0.93	*0.15*	1.36	*0.20*	1.27	*0.16*	1.11	*0.25*
6.95E-08	**hsa-miR-744**	1.84	*0.07*	1.09	*0.06*	2.03	*0.01*	0.98	*0.02*	0.80	*0.00*	1.16	*0.04*	1.74	*0.04*	1.22	*0.01*	2.26	*0.06*	1.29	*0.03*	1.38	*0.07*
9.74E-10	**hsa-miR-765**	1.33	*0.02*	0.98	*0.00*	1.31	*0.01*	0.91	*0.05*	0.86	*0.00*	0.96	*0.10*	1.17	*0.01*	1.04	*0.01*	1.29	*0.01*	1.17	*0.00*	1.11	*0.04*
1.39E-09	**hsa-miR-768-3p**	1.49	*0.01*	0.94	*0.01*	1.49	*0.01*	1.13	*0.06*	1.09	*0.01*	1.16	*0.12*	1.15	*0.09*	0.71	*0.03*	1.59	*0.14*	0.92	*0.06*	1.29	*0.06*
1.09E-05	**hsa-miR-768-5p**	0.97	*0.06*	0.42	*0.09*	1.05	*0.10*	0.75	*0.01*	0.67	*0.09*	0.84	*0.05*	0.67	*0.14*	0.42	*0.06*	0.93	*0.18*	0.52	*0.08*	0.80	*0.17*
8.7E-09	**hsa-miR-877**	2.52	*0.04*	1.34	*0.09*	3.43	*0.02*	1.22	*0.09*	0.75	*0.14*	1.66	*0.20*	2.38	*0.11*	1.68	*0.01*	3.06	*0.18*	2.09	*0.01*	1.12	*0.10*
1.88E-15	**hsa-miR-886-3p**	2.52	*0.22*	1.90	*0.21*	0.89	*0.20*	1.26	*0.22*	1.86	*0.17*	0.66	*0.36*	1.22	*0.20*	1.38	*0.18*	1.06	*0.21*	3.51	*0.16*	1.83	*0.12*
5.88E-11	**hsa-miR-886-5p**	2.10	*0.08*	1.07	*0.15*	0.95	*0.21*	0.82	*0.08*	0.94	*0.21*	0.69	*0.09*	1.04	*0.09*	0.99	*0.07*	1.07	*0.24*	2.36	*0.08*	1.35	*0.01*
3.39E-10	**hsa-miR-888***	1.52	*0.09*	1.08	*0.05*	1.50	*0.07*	1.19	*0.01*	0.62	*0.13*	1.75	*0.03*	1.01	*0.07*	0.84	*0.07*	1.18	*0.06*	1.73	*0.07*	1.27	*0.08*
9.01E-16	**hsa-miR-891a**	2.48	*0.12*	1.85	*0.05*	2.10	*0.17*	1.71	*0.17*	2.00	*0.15*	1.42	*0.19*	1.61	*0.18*	1.12	*0.13*	2.09	*0.21*	3.55	*0.14*	1.74	*0.19*
5.64E-09	**hsa-miR-923**	2.41	*0.02*	2.12	*0.06*	2.34	*0.02*	1.99	*0.10*	2.42	*0.06*	1.56	*0.17*	2.68	*0.02*	2.85	*0.03*	2.50	*0.07*	1.58	*0.01*	2.83	*0.05*
8.8E-11	**hsa-miR-92a**	1.07	*0.01*	0.65	*0.05*	1.17	*0.03*	0.54	*0.04*	0.41	*0.03*	0.66	*0.04*	0.92	*0.07*	0.58	*0.03*	1.27	*0.12*	0.84	*0.00*	0.87	*0.04*
4.53E-12	**hsa-miR-92b**	1.06	*0.04*	0.64	*0.09*	1.19	*0.06*	0.55	*0.03*	0.41	*0.04*	0.69	*0.02*	0.98	*0.04*	0.59	*0.05*	1.38	*0.04*	0.82	*0.04*	0.83	*0.00*
3.4E-15	**hsa-miR-93**	1.29	*0.02*	0.84	*0.03*	1.39	*0.01*	0.70	*0.10*	0.28	*0.04*	1.12	*0.14*	0.91	*0.01*	0.57	*0.01*	1.24	*0.02*	1.33	*0.00*	1.46	*0.02*
2.86E-18	**hsa-miR-933**	1.83	*0.02*	1.32	*0.01*	1.75	*0.00*	1.31	*0.00*	1.17	*0.00*	1.46	*0.00*	1.63	*0.00*	1.33	*0.03*	1.92	*0.02*	1.84	*0.03*	1.44	*0.02*
8.27E-12	**hsa-miR-939**	2.15	*0.02*	1.29	*0.02*	2.47	*0.06*	-	-	1.12	*0.02*	1.71	*0.07*	1.96	*0.03*	1.45	*0.00*	2.47	*0.05*	1.59	*0.01*	1.63	*0.04*
