# Supplementary material for: Expression Profile of MicroRNAs in Young Stroke Patients
Source: PLoS One. 2009 Nov 2;4(11):e7689. doi: 10.1371/journal.pone.0007689 (PMC2765616; doi:10.1371/journal.pone.0007689)
Supplement: Table S2 — miRNA that showed mixed (up/and/or down) expression in stroke. Only fold change is shown (refer to Table S1 for ?SEM). The miRNAs that are upregulated in stroke (n = 19) is marked in red and the downregulated miRNAs are in green fonts. (0.07 MB PDF) [file pone.0007689.s002.pdf]

miRNA	Stroke	Stroke mRS<2	Stroke mRS>2	LA	LA mRS<2	LA mRS>2	CEmb	Cemb mRS<2	Cemb mRS>2	SA	UND
hsa-let-7a	0.76	0.33	0.71	0.39	0.13	0.65	0.38	0.18	0.57	0.68	1.01
hsa-let-7b	1.23	0.58	1.30	0.51	0.27	0.75	0.87	0.51	1.23	1.13	1.12
hsa-let-7c	1.18	0.59	1.29	0.55	0.29	0.81	0.85	0.52	1.19	1.10	1.12
hsa-let-7d	0.75	0.39	0.82	0.46	0.16	0.76	0.42	0.19	0.64	0.70	1.08
hsa-let-7d*	1.59	0.67	1.68	0.77	0.36	1.18	1.21	0.83	1.59	1.02	0.86
hsa-let-7g	0.93	0.44	0.88	0.45	0.13	0.77	0.44	0.24	0.64	0.73	1.24
hsa-let-7i	0.91	0.44	0.84	0.49	0.12	0.86	0.44	0.20	0.67	0.83	1.33
hsa-miR-101	1.54	0.87	1.47	0.83	0.31	1.34	0.88	0.54	1.22	1.98	2.05
hsa-miR-103	1.31	0.82	1.40	0.84	0.27	1.41	0.93	0.64	1.21	1.64	1.43
hsa-miR-106a	1.49	1.02	1.51	0.84	0.22	1.45	0.84	0.50	1.18	1.77	1.82
hsa-miR-106b	1.42	0.90	1.51	0.77	0.23	1.31	1.02	0.68	1.35	1.77	1.39
hsa-miR-106b*	1.42	0.73	1.52	0.80	0.36	1.25	1.03	0.75	1.31	1.37	1.30
hsa-miR-107	1.17	0.64	1.22	0.76	0.22	1.30	0.72	0.51	0.92	1.37	1.29
hsa-miR-1255a	1.03	0.68	1.25	0.73	0.58	0.87	0.90	0.59	1.21	0.95	1.11
hsa-miR-125b-1*	1.98	1.29	2.58	1.15	0.96	1.33	2.07	1.00	3.14	1.90	1.70
hsa-miR-1264	1.53	1.03	1.87	1.03	0.32	1.74	1.21	0.76	1.65	1.64	1.49
hsa-miR-1265	1.36	0.75	1.20	0.99	0.70	1.28	1.00	0.80	1.20	1.13	1.18
hsa-miR-1280	1.99	1.67	2.14	1.10	0.96	1.24	1.72	1.47	1.98	2.22	2.09
hsa-miR-1284	1.01	0.59	1.07	0.66	0.48	0.84	0.66	0.37	0.95	0.84	0.94
hsa-miR-1285	1.87	1.22	1.68	1.34	1.54	1.14	1.37	1.04	1.70	1.30	1.39
hsa-miR-1290	2.01	1.70	1.98	2.14	2.71	1.57	2.27	2.20	2.33	1.37	2.27
hsa-miR-129-5p	1.73	1.13	2.07	1.00	0.93	1.07	1.95	1.00	2.89	1.52	1.54
hsa-miR-1308	1.03	0.57	0.98	0.90	0.77	1.03	0.75	0.64	0.86	0.63	0.67
hsa-miR-130a	1.56	0.80	1.64	0.99	0.30	1.69	0.92	0.62	1.21	1.70	1.35
hsa-miR-130b	1.78	1.17	1.90	0.89	0.30	1.47	1.35	1.06	1.65	2.33	1.84
hsa-miR-138-1*	1.20	0.94	1.20	0.93	0.35	1.51	0.98	0.66	1.29	1.43	1.20
hsa-miR-140-3p	1.29	0.76	1.20	0.72	0.29	1.15	0.89	0.73	1.05	1.12	0.99
hsa-miR-142-5p	1.42	0.86	1.32	0.80	0.27	1.32	0.85	0.55	1.14	1.24	1.54
hsa-miR-144	1.35	0.87	1.61	0.85	0.42	1.28	1.17	0.65	1.70	1.70	1.57
hsa-miR-144*	0.69	1.30	0.71	0.60	0.06	1.14	0.32	0.05	0.59	1.07	1.43
hsa-miR-148b	1.24	0.70	1.24	0.56	0.16	0.95	0.77	0.50	1.04	0.91	1.16
hsa-miR-149*	1.68	1.01	2.25	1.18	0.78	1.58	1.94	0.99	2.89	1.21	1.38
hsa-miR-150	2.22	0.69	1.62	0.61	0.50	0.72	0.84	0.52	1.16	1.01	1.62
hsa-miR-151-3p	2.38	1.34	2.48	1.00	0.29	1.70	1.71	1.08	2.34	2.65	2.40
hsa-miR-151-5p	1.47	0.84	1.33	0.75	0.12	1.38	0.66	0.28	1.04	1.56	1.78
hsa-miR-15a	1.07	0.57	1.18	0.63	0.24	1.03	0.66	0.36	0.96	1.21	1.29
hsa-miR-16	1.02	0.62	1.03	0.67	0.30	1.03	0.74	0.46	1.02	1.03	1.01
hsa-miR-16-2*	1.42	0.70	1.82	0.84	0.09	1.60	1.01	0.38	1.61	1.38	1.82
hsa-miR-17	1.26	0.79	1.26	0.72	0.19	1.26	0.67	0.41	0.94	1.48	1.47
hsa-miR-17*	2.02	0.84	1.80	0.73	0.29	1.15	1.41	0.94	1.88	1.63	1.70
hsa-miR-182	1.35	0.59	1.13	0.48	0.09	0.86	0.68	0.46	0.89	1.37	1.39
hsa-miR-1826	1.01	0.61	0.97	0.91	0.89	0.94	0.74	0.59	0.89	0.71	0.88
hsa-miR-1827	1.24	0.72	1.15	0.85	0.76	0.94	1.03	0.91	1.15	0.82	1.23
hsa-miR-183	1.32	0.78	1.14	0.54	0.23	0.84	0.99	0.83	1.15	1.22	1.10
hsa-miR-183*	1.88	0.96	2.27	1.02	0.84	1.20	1.83	0.76	2.89	1.59	1.34
hsa-miR-184	1.93	1.75	2.82	1.33	0.99	1.67	2.29	1.45	3.14	2.43	2.16
hsa-miR-185	1.78	0.91	1.90	0.93	0.25	1.62	1.10	0.92	1.28	1.54	1.39
hsa-miR-185*	2.15	1.15	2.93	1.03	0.82	1.25	2.32	0.95	3.70	1.42	1.75
hsa-miR-18a	1.96	1.22	2.13	1.09	0.24	1.93	1.00	0.68	1.32	2.74	2.36
hsa-miR-18b	1.46	1.06	1.59	0.87	0.21	1.53	0.81	0.51	1.11	1.90	1.86
hsa-miR-191	1.47	0.92	1.47	0.70	0.32	1.09	1.06	0.87	1.25	1.34	1.33
hsa-miR-194	1.79	0.84	1.93	0.87	0.20	1.53	1.05	0.76	1.34	1.64	1.39
hsa-miR-195	1.06	0.27	1.01	0.45	0.08	0.82	0.45	0.16	0.73	1.32	1.24
hsa-miR-19a	1.41	0.81	1.33	0.82	0.30	1.35	0.87	0.51	1.22	1.23	1.49

hsa-miR-19b	1.65	1.10	1.47	0.88	0.52	1.24	1.18	0.86	1.49	1.62	1.74
hsa-miR-20b	1.29	0.71	1.29	0.69	0.12	1.25	0.65	0.36	0.93	1.37	1.46
hsa-miR-21	1.34	0.58	1.28	0.75	0.17	1.33	0.75	0.35	1.15	1.72	1.79
hsa-miR-22	1.26	0.85	1.28	0.59	0.30	0.87	1.17	1.03	1.30	0.99	1.00
hsa-miR-222	1.31	0.85	1.26	0.67	0.17	1.18	0.83	0.56	1.11	1.70	1.57
hsa-miR-223	1.44	0.88	1.42	0.93	0.25	1.61	0.57	0.35	0.80	1.71	1.72
hsa-miR-23a	1.63	1.21	1.37	0.86	0.37	1.36	0.80	0.61	0.97	1.78	1.94
hsa-miR-23b	1.37	0.97	1.18	0.67	0.21	1.10	0.78	0.64	0.91	1.39	1.75
hsa-miR-24	1.23	0.87	1.06	0.58	0.25	0.91	0.71	0.60	0.82	1.17	1.50
hsa-miR-24-1*	1.19	0.97	1.30	0.81	0.39	1.24	1.27	0.83	1.72	1.24	1.27
hsa-miR-25	1.24	0.80	1.21	0.56	0.27	0.85	0.80	0.52	1.08	1.23	1.44
hsa-miR-26a	1.17	0.70	1.10	0.58	0.18	0.98	0.67	0.43	0.91	1.28	1.29
hsa-miR-26b	0.97	0.47	0.92	0.57	0.19	0.94	0.48	0.28	0.68	1.00	1.19
hsa-miR-299-3p	2.15	1.45	1.79	1.16	1.41	0.90	1.84	1.39	2.29	1.71	1.27
hsa-miR-29a	1.40	0.85	1.48	0.75	0.28	1.22	0.96	0.64	1.29	1.34	1.42
hsa-miR-29b	1.58	1.10	1.45	0.84	0.44	1.23	0.99	0.41	1.56	2.03	1.77
hsa-miR-29c	2.34	1.21	2.38	1.08	0.30	1.85	1.58	1.01	2.13	3.14	2.25
hsa-miR-300	1.28	0.84	1.11	0.56	0.56	0.56	1.10	0.78	1.42	1.31	1.29
hsa-miR-301a	1.56	0.88	1.63	0.97	0.20	1.74	0.81	0.47	1.15	2.05	2.07
hsa-miR-30a	1.30	0.86	1.41	0.72	0.34	1.11	1.05	0.72	1.38	1.33	1.34
hsa-miR-30b	0.98	0.57	1.00	0.59	0.16	1.01	0.53	0.28	0.78	1.07	1.24
hsa-miR-30c	1.29	0.73	1.17	0.66	0.17	1.16	0.64	0.39	0.89	1.30	1.53
hsa-miR-30d	1.48	0.99	1.66	0.77	0.39	1.16	1.36	1.03	1.68	1.29	1.22
hsa-miR-30e	1.16	0.74	1.18	0.71	0.22	1.20	0.73	0.40	1.05	1.21	1.47
hsa-miR-32*	1.04	0.48	1.15	0.66	0.54	0.78	0.75	0.56	0.95	0.77	0.76
hsa-miR-320a	2.07	1.19	2.60	1.10	0.70	1.50	2.18	1.57	2.80	1.75	1.35
hsa-miR-320b	1.52	0.88	1.81	0.86	0.57	1.15	1.53	1.19	1.87	1.26	1.03
hsa-miR-320c	1.39	0.73	1.77	0.78	0.47	1.09	1.40	1.00	1.79	1.04	0.84
hsa-miR-320d	1.56	0.88	1.96	0.87	0.60	1.13	1.55	1.14	1.97	1.28	1.04
hsa-miR-324-5p	1.26	0.43	1.14	0.44	0.15	0.73	0.65	0.46	0.83	0.84	0.70
hsa-miR-326	1.31	0.96	1.24	1.00	0.68	1.31	0.74	0.49	0.99	1.63	1.33
hsa-miR-331-3p	1.44	0.72	1.71	0.89	0.41	1.37	1.27	0.94	1.60	1.15	0.95
hsa-miR-339-5p	2.26	1.40	1.86	0.98	0.48	1.48	1.74	1.78	1.71	2.08	1.75
hsa-miR-342-3p	1.29	0.66	1.13	0.65	0.15	1.15	0.56	0.36	0.75	0.83	1.39
hsa-miR-34b	1.20	0.88	1.38	1.03	0.99	1.06	1.14	0.53	1.75	1.08	1.15
hsa-miR-361-5p	1.58	1.06	1.79	0.88	0.23	1.52	1.23	0.88	1.54	1.80	2.05
hsa-miR-362-5p	1.78	0.78	1.85	0.75	0.18	1.31	1.12	0.55	1.67	1.73	1.54
hsa-miR-363	1.24	0.57	1.42	0.67	0.24	1.09	0.95	0.57	1.33	1.10	1.10
hsa-miR-374a	0.93	0.50	0.96	0.66	0.16	1.16	0.47	0.25	0.68	1.00	1.11
hsa-miR-378	1.43	0.82	1.30	0.63	0.25	1.01	1.14	0.96	1.31	1.22	1.23
hsa-miR-423-3p	1.35	0.77	1.44	0.76	0.44	1.07	1.21	1.11	1.32	0.96	0.90
hsa-miR-423-5p	1.81	0.86	2.22	0.91	0.68	1.14	1.57	1.18	1.96	1.11	0.92
hsa-miR-425	1.29	0.91	1.41	0.82	0.44	1.19	1.15	0.80	1.50	1.19	1.45
hsa-miR-451	1.00	1.00	1.00	1.00	1.00	1.00	1.00	1.00	1.00	1.00	1.00
hsa-miR-484	1.49	0.86	1.61	0.82	0.53	1.11	1.32	1.03	1.61	0.99	0.90
hsa-miR-485-3p	1.53	0.78	1.74	0.98	0.34	1.62	1.43	0.78	2.08	1.00	1.65
hsa-miR-486-5p	1.21	1.19	1.21	1.06	0.90	1.21	1.21	1.21	1.21	1.08	1.19
hsa-miR-487b	1.27	0.79	1.32	0.76	0.17	1.34	0.99	0.64	1.34	1.62	0.90
hsa-miR-491-3p	1.57	1.25	1.58	1.01	0.80	1.23	1.47	1.40	1.54	1.37	1.16
hsa-miR-500	2.07	1.50	2.72	1.41	0.31	2.51	1.81	1.24	2.39	2.81	3.41
hsa-miR-501-5p	1.52	0.65	1.69	0.74	0.30	1.17	1.27	0.76	1.77	1.20	0.70
hsa-miR-519d	1.78	1.29	2.10	1.22	0.36	2.09	1.29	1.03	1.56	2.21	1.98
hsa-miR-519e	0.63	0.59	0.90	0.78	0.58	0.97	0.43	0.30	0.57	0.93	0.76
hsa-miR-519e*	2.76	2.04	3.53	2.18	1.25	3.11	2.77	1.75	3.79	3.23	2.86
hsa-miR-532-5p	1.65	0.83	1.74	0.66	0.29	1.02	1.07	0.54	1.59	2.10	2.07
hsa-miR-549	1.65	1.26	1.72	1.21	1.23	1.19	1.62	0.99	2.25	1.74	1.52

**hsa-miR-550***	**1.37**	0.94	1.50	0.85	0.41	1.29	1.26	1.04	1.47	1.27	1.25
**hsa-miR-551b**	**1.12**	0.85	1.13	0.89	0.89	0.89	0.84	0.66	1.02	1.05	1.25
**hsa-miR-574-5p**	**1.05**	0.38	1.32	0.56	0.50	0.62	0.74	0.43	1.04	0.74	0.63
**hsa-miR-584**	**1.87**	1.20	2.03	1.16	0.70	1.62	1.48	0.95	2.02	2.39	1.80
**hsa-miR-628-3p**	**1.67**	0.94	2.30	0.96	0.87	1.04	2.09	0.77	3.41	1.33	1.23
**hsa-miR-634**	**1.70**	1.49	2.31	0.94	0.57	1.31	1.41	1.00	1.81	2.72	1.87
**hsa-miR-637**	**2.25**	1.30	2.96	1.13	1.10	1.16	2.43	0.95	3.92	2.00	1.87
**hsa-miR-638**	**1.52**	1.16	2.36	1.10	1.09	1.10	2.23	0.92	3.54	1.30	1.62
**hsa-miR-652**	**1.14**	0.66	1.30	0.71	0.43	0.99	1.03	0.72	1.33	1.01	0.88
**hsa-miR-668**	**1.96**	0.97	2.09	1.25	1.05	1.44	1.33	0.70	1.97	1.91	1.70
**hsa-miR-7**	**0.81**	0.34	0.86	0.48	0.25	0.72	0.47	0.23	0.71	0.93	1.58
**hsa-miR-720**	**1.63**	0.91	1.51	0.96	0.65	1.27	1.15	0.93	1.36	1.27	1.11
**hsa-miR-744**	**1.84**	1.09	2.03	0.98	0.80	1.16	1.74	1.22	2.26	1.29	1.38
**hsa-miR-765**	**1.33**	0.98	1.31	0.91	0.86	0.96	1.17	1.04	1.29	1.17	1.11
**hsa-miR-768-3p**	**1.49**	0.94	1.49	1.13	1.09	1.16	1.15	0.71	1.59	0.92	1.29
**hsa-miR-877**	**2.52**	1.34	3.43	1.22	0.75	1.66	2.38	1.68	3.06	2.09	1.12
**hsa-miR-886-3p**	**2.52**	1.90	0.89	1.26	1.86	0.66	1.22	1.38	1.06	3.51	1.83
**hsa-miR-886-5p**	**2.10**	1.07	0.95	0.82	0.94	0.69	1.04	0.99	1.07	2.36	1.35
**hsa-miR-888***	**1.52**	1.08	1.50	1.19	0.62	1.75	1.01	0.84	1.18	1.73	1.27
**hsa-miR-92a**	**1.07**	0.65	1.17	0.54	0.41	0.66	0.92	0.58	1.27	0.84	0.87
**hsa-miR-92b**	**1.06**	0.64	1.19	0.55	0.41	0.69	0.98	0.59	1.38	0.82	0.83
**hsa-miR-93**	**1.29**	0.84	1.39	0.70	0.28	1.12	0.91	0.57	1.24	1.33	1.46
