# Supplementary material for: Expression Profile of MicroRNAs in Young Stroke Patients
Source: PLoS One. 2009 Nov 2;4(11):e7689. doi: 10.1371/journal.pone.0007689 (PMC2765616; doi:10.1371/journal.pone.0007689)
Supplement: Table S4 — miRNAs that are differentially expressed in stroke patients and the pathways that they possibly regulate. The miRNA:biological process relationship was predicted using miRNApath search tool (http://lgmb.fmrp.usp.br/mirnapath/tools.php and http://diana.cslab.ece.ntua.gr/) [30]. The pathways that have the largest number of genes affected by the miRNAs and with the highest [−ln(p-value)] are listed. (0.07 MB DOC) [file pone.0007689.s004.doc]

**Table S4**

| **Affected by microRNAs that are upregulated in stroke** | | | | **Affected by microRNAs that are downregulated in stroke** | | | |
| --- | --- | --- | --- | --- | --- | --- | --- |
| **KEGG Pathway** | **KEGG ID** | **Found Genes** | **-ln(p-value)** | **KEGG Pathway** | **KEGG ID** | **Found Genes** | **-ln(p-value)** |
| MAPK signaling pathway | hsa04010 | 58 | 8.8 | MAPK signaling pathway | hsa04010 | 57 | 17.32 |
| Focal adhesion | hsa04510 | 55 | 16.87 | Axon guidance | hsa04360 | 30 | 10.69 |
| TGF-beta signaling pathway | hsa04350 | 32 | 16.46 | Focal adhesion | hsa04510 | 39 | 8.42 |
| Wnt signaling pathway | hsa04310 | 41 | 11.87 | Wnt signaling pathway | hsa04310 | 29 | 5.87 |
| Regulation of actin cytoskeleton | hsa04810 | 53 | 11.62 | TGF-beta signaling pathway | hsa04350 | 27 | 15.53 |
| VEGF signaling pathway | hsa04370 | 23 | 9.74 | Jak-STAT signaling pathway | hsa04630 | 25 | 2.44 |
| Adherens junction | hsa04520 | 23 | 9.09 | Calcium signaling pathway | hsa04020 | 25 | 1.61 |
| Ubiquitin mediated proteolysis | hsa04120 | 35 | 8.9 | Cytokine-cytokine receptor interaction | hsa04060 | 37 | 1.75 |
| Axon guidance | hsa04360 | 31 | 6.29 | Regulation of actin cytoskeleton | hsa04810 | 35 | 3.84 |
| Insulin signaling pathway | hsa04910 | 33 | 5.64 | T cell receptor signaling pathway | hsa04660 | 20 | 5.62 |
| Cell cycle | hsa04110 | 28 | 5.44 | Insulin signaling pathway | hsa04910 | 26 | 4.34 |
| Calcium signaling pathway | hsa04020 | 37 | 5.04 | Ubiquitin mediated proteolysis | hsa04120 | 20 | 1.4 |
| T cell receptor signaling pathway | hsa04660 | 22 | 4.1 | Tight junction | hsa04530 | 20 | 1.16 |
| Jak-STAT signaling pathway | hsa04630 | 33 | 3.9 |  |  |  |  |
| ErbB signaling pathway | hsa04012 | 21 | 3.61 |  |  |  |  |
| Toll-like receptor signaling pathway | hsa04620 | 22 | 2.6 |  |  |  |  |
| Gap junction | hsa04540 | 20 | 2.17 |  |  |  |  |
| Tight junction | hsa04530 | 25 | 1.35 |  |  |  |  |
| Neuroactive ligand-receptor interaction | hsa04080 | 33 | 0.78 |  |  |  |  |
| Cytokine-cytokine receptor interaction | hsa04060 | 42 | 0.75 |  |  |  |  |
| Natural killer cell mediated cytotoxicity | hsa04650 | 20 | 0.33 |  |  |  |  |
|  |  |  |  |  |  |  |  |
