# Supplementary material for: Expression Profile of MicroRNAs in Young Stroke Patients
Source: PLoS One. 2009 Nov 2;4(11):e7689. doi: 10.1371/journal.pone.0007689 (PMC2765616; doi:10.1371/journal.pone.0007689)
Supplement: Table S5 — Patient profile and medications (a) Demographic comparison and (b) Detailed medication and modified Rankin Score of young Asian stroke patients selected for the study. Controls (n = 5) included 3 males and 2 females. (0.07 MB DOC) [file pone.0007689.s005.doc]

Table S5: **a)** Demographic data of the young Asian stroke patients selected for the study. Controls (n=5) included 3 males and 2 females. **b)** Detailed list of medications and modified Rankin Score descriptions

for each stroke patient that was analysed in our study.

| **sample** | **Stroke classification** |  | **Dich Outcome** | **Prescription** |
| --- | --- | --- | --- | --- |
| **code** |  | **Rankin (mRS)** | **0-2 good = 1** |  |
|  |  |  | **3-6 poor = 0** |  |
| BE | Cardioembolic (CEmb) | 2 | good | Warfarin, Caspirin, Simvastatin |
| BF | Cardioembolic | 1 | good | Atenolol, Frusemide, Simvastatin, Warfarin, Losartan |
| AF | Cardioembolic | 2 | good | Caspirin, Warfarin, Metformin, Simvastatin, Losartan, hydrochlorothiazide (HCT), amlodipine, gliclazide |
| BG | Cardioembolic | 3 | poor | Simavastatin, Metoprolol, Warfarin, perindropril, Metformin, Diamicron |
| BA | Cardioembolic | 3 | poor | Amlodipine, Caspirin, Simvastatin |
| A | Large artery (LA) | 1 | good | Perindopril, Simvastatin, Caspirin |
| C | Large artery | 2 | good | Caspirin, Simvastatin, Amlodipine, HCT, Perindopril, Dipyridamole |
| DD | Large artery | 2 | good | Caspirin, Simvastatin, Folic Acid, Vitamin B12, Gliclazide, Metformin, perindropril, Atenolol, HCT |
| E | Large artery | 2 | good | Caspirin, Simvastatin, Losartan |
| AB | Large artery | 0 | good | HCT, Atorvastatin, Gliclazide, Dipyridamole, Metformin, Caspirin, Irbesartan |
| BB | Large artery | 1 | good | Caspirin |
| DB | Large artery | 4 | poor | Warfarin, Coversyl |
| F | Large artery | 3 | poor | Telmisartan, Metformin, Actrapid, Insulatard, Atenolol, Norvasc, Caspirin, Simvastatin |
| D | Small artery (SA) | 2 | good | Acarbose, Metformin, Atorvastatin, Caspirin, Losartan, HCT, Metoprolol, frusemide, Isordil |
| AC | Small artery | 0 | good | Gliclazide, perindropril, Simvastatin, Ticlopidine, Metformin, Fenofibrate |
| AD | Small artery | 1 | good | Perindropril, Amlodipine, Aspirin, Simvastatin |
| AA | Undetermined (UND) | 2 | good | Telmisartan, Dipyridamole, Caspirin, Simastatin, HCT, Glibendamide, Meformin |
| BD | Undetermined | 1 | good | Perindopril, Aspirin, Simvastatin |
| B | Undetermined | 1 | good | Valsartan, Atenolol, Hydrochlorothiazide, Gliclazide, Amlodopine, Metformin, Simvastatin, Caspirin |

b)

| **a)** |  | **Large artery** | **Lacunar** | **Cardioembolic** | **Undetermined** |
| --- | --- | --- | --- | --- | --- |
| **Gender** | male (M) | 6 | 1 | 2 | 1 |
|  | female (F) | 2 | 2 | 3 | 2 |
| **Age years (mean)±** |  | 38.57±10.65 | 37.67±8.33 | 36.00±0.0 | 43.33±3.52 |
| **Risk factors** |  |  |  |  |  |
| **Cigarette smoking** |  | 1M | 1M | 1M | 1F |
| **Hypertension** |  | 4M, 1F | 2M | 2F, 1M | 1M, 1F |
| **Diabetes** |  | 3M, 1F | 1F,1M | 2M | 1M,1F |
| **Hypercholesterolimia** |  | 4M,1F | 1M,1F | 1F,1M | 1M |
| **Alcohol** |  | - | 1M | - | - |
|  |  |  |  |  |  |
| **Sample ID** |  | A, C,E,F, AB,BB,DB,DD, | D,AC,AD | AF,BA,BE,BF,BG | B, AA,BD |
